# Unraveling pathways of elevated ozone induced by the 2020 lockdown in Europe by an observationally constrained regional model using TROPOMI

**DOI:** 10.5194/acp-21-18227-2021

**Published:** 2021-12-16

**Authors:** Amir H. Souri, Kelly Chance, Juseon Bak, Caroline R. Nowlan, Gonzalo González Abad, Yeonjin Jung, David C. Wong, Jingqiu Mao, Xiong Liu

**Affiliations:** 1 Atomic and Molecular Physics (AMP) Division, Harvard–Smithsonian Center for Astrophysics, Cambridge, MA, USA; 2 Institute of Environmental Studies, Pusan National University, Busan, South Korea; 3 US Environmental Protection Agency, Center for Environmental Measurement & Modeling, Research Triangle Park, NC, USA; 4 Geophysical Institute, University of Alaska Fairbanks, Fairbanks, AK, USA; 5 Department of Chemistry and Biochemistry, University of Alaska Fairbanks, Fairbanks, AK, USA

## Abstract

Questions about how emissions are changing during the COVID-19 lockdown periods cannot be answered by observations of atmospheric trace gas concentrations alone, in part due to simultaneous changes in atmospheric transport, emissions, dynamics, photochemistry, and chemical feedback. A chemical transport model simulation benefiting from a multi-species inversion framework using well-characterized observations should differentiate those influences enabling to closely examine changes in emissions. Accordingly, we jointly constrain NO_*x*_ and VOC emissions using well-characterized TROPOspheric Monitoring Instrument (TROPOMI) HCHO and NO_2_ columns during the months of March, April, and May 2020 (lockdown) and 2019 (baseline). We observe a noticeable decline in the magnitude of NO_*x*_ emissions in March 2020 (14 %–31 %) in several major cities including Paris, London, Madrid, and Milan, expanding further to Rome, Brussels, Frankfurt, Warsaw, Belgrade, Kyiv, and Moscow (34 %–51 %) in April. However, NO_*x*_ emissions remain at somewhat similar values or even higher in some portions of the UK, Poland, and Moscow in March 2020 compared to the baseline, possibly due to the timeline of restrictions. Comparisons against surface monitoring stations indicate that the constrained model underrepresents the reduction in surface NO_2_. This underrepresentation correlates with the TROPOMI frequency impacted by cloudiness. During the month of April, when ample TROPOMI samples are present, the surface NO_2_ reductions occurring in polluted areas are described fairly well by the model (model: −21 ± 17 %, observation: −29 ± 21 %). The observational constraint on VOC emissions is found to be generally weak except for lower latitudes. Results support an increase in surface ozone during the lockdown. In April, the constrained model features a reasonable agreement with maximum daily 8 h average (MDA8) ozone changes observed at the surface (*r* = 0.43), specifically over central Europe where ozone enhancements prevail (model: +3.73 ± 3.94 %, + 1.79 ppbv, observation: +7.35 ± 11.27 %, +3.76 ppbv). The model suggests that physical processes (dry deposition, advection, and diffusion) decrease MDA8 surface ozone in the same month on average by −4.83 ppbv, while ozone production rates dampened by largely negative $J_{{\text{NO}}_{2}}\left[{\text{NO}}_{2}\right]-k_{\text{NO}+{\text{O}}_{3}}[\text{NO}]\left[{\text{O}}_{3}\right]$ become less negative, leading ozone to increase by +5.89 ppbv. Experiments involving fixed anthropogenic emissions suggest that meteorology contributes to 42 % enhancement in MDA8 surface ozone over the same region with the remaining part (58 %) coming from changes in anthropogenic emissions. Results illustrate the capability of satellite data of major ozone precursors to help atmospheric models capture ozone changes induced by abrupt emission anomalies.

## 1 Introduction

Continuous monitoring of air pollution by satellites can help our understanding of both anthropogenic and biogenic variability and change caused by rapid economic recession (Castellanos and Boersma, 2012) and regulations (Krotkov et al., 2016; Souri et al., 2020a). Earth’s atmosphere has substantially become more polluted since the industrial era in comparison to its original environmental condition (Li and Lin, 2015); thus, any abrupt hiatus in anthropogenic (manmade) emissions should result in an immediate impact on relatively short lifetime pollutants such as nitrogen dioxide (NO_2_), formaldehyde (HCHO), and tropospheric ozone (O_3_). The beginning of the global COVID-19 pandemic in early 2020 (Fauci et al., 2020) provided such an abrupt change in human activities (Le Quéré et al., 2020). A first step to fully understand how much of these impacts are related to the pandemic lockdowns is to disentangle the physiochemical processes determining their ambient concentrations. Unraveling those processes requires precise, continuous observations of physical and chemical states and emission rates, which are not routinely available on global, continental, and regional scales. Therefore, we resort to using a model realization attempting to reproduce such an intricate system. Models without observational guidance are incapable of numerically representing the real world (Lorenz, 1963), so our best option to improve a model is to constrain some of its prognostic inputs using well-characterized observations. Accordingly, the framework of this study is centered around inverse modeling and data assimilation.

Significant attention has been given to documenting the lockdown-related changes in atmospheric composition around the world using both in situ and satellite observations (e.g., Sicard et al., 2020; Shi and Brasseur, 2020; Lee et al., 2020; Salma et al., 2020; Le Quéré et al., 2020; He et al., 2020; Le et al., 2020; Miyazaki et al., 2020; Liu et al., 2020; Barré et al., 2021; Goldberg et al., 2020; Ordóñez et al., 2020; Wyche et al., 2021; Bekbulat et al., 2020; Gaubert et al., 2021; Sun et al., 2021). The broad picture is consistent among these studies; the lockdown drastically reduced the concentrations of NO_*x*_, CO, and SO_2_ and some types of particulate matter, whereas the concentrations of several secondarily formed compounds such as ozone behaved in non-linear ways due to emissions and/or meteorology.

The motivations of this study are to determine the capability of a regional model constrained by satellite HCHO and NO_2_ columns to capture near-surface pollution and whether the local ozone production rates are the driving factors for heightening ozone pollution during the 2020 lockdown. In other words, what physiochemical processes are associated with the elevated ozone? How representative are satellite observations of captured surface air quality through an inversion context? Is meteorology the primary factor in shaping elevated ozone, as suggested by Ordóñez et al. (2020)?

To address these pivotal questions, it is desirable to constrain models using multi-species observations because relationships between the atmospheric compounds such as HCHO and NO_2_ are importantly intertwined (Marais et al., 2012; Valin et al., 2016; Wolfe et al., 2016; Souri et al., 2020a, b). Accordingly, we build our inversion framework upon a non-linear joint analytical inversion of NO_*x*_ and VOCs proposed in Souri et al. (2020a) using the TROPOspheric Monitoring Instrument (TROPOMI) HCHO and NO_2_ observations in Europe. Performing this type of inversion not only enables us to precisely quantify the changes in emissions (along with its uncertainty, as the inversion framework is analytical) but also paves the way for estimating the resulting changes on different pathways of ozone.

## 2 Measurements, modeling, and methods

### 2.1 Satellite observations

#### 2.1.1 TROPOMI NO_2_

We use daily offline S5P TROPOMI tropospheric NO_2_ slant columns (Copernicus Sentinel data processed by ESA and Koninklijk Nederlands Meteorologisch Instituut (KNMI), 2019) derived from a two-step framework involving differential optical absorption spectroscopy (DOAS) spectral fitting in conjunction with a stratosphere–troposphere decoupler (Boersma et al., 2018). The time periods of this study are March, April, and May 2020 and 2019. The data provide Jacobians of light intensity with respect to optical thickness (i.e., vertically resolved scattering weights), which are dependent on scene surface reflectivity, the cloudiness of the assumed Lambertian clouds, and the sensor viewing geometry.

Aerosol effects on the scattering weights are not taken into consideration. Based on radiative transfer calculations and satellite-based aerosol products, Y. Jung et al. (2019) and Cooper et al. (2019) observed small changes (< 10 %) in air mass factors (AMFs) with and without considering the aerosol impacts in Europe in springtime. This tendency likely results from a low aerosol optical depth.

The 2019 TROPOMI observations used in this study have a spatial resolution of 7 × 3.5 km^2^, whereas those in 2020 have a spatial resolution of 5.5 × 3.5 km^2^. The NO_2_ products for the study time period were produced by processor versions v01.02.02 (1–20 March 2019) and v01.03.02 (20 March 2019 onward). The v01.03.02 processor includes an update to the FRESCO-S cloud algorithm and improvements to a quality flag variable. NO_2_ validation from processors v01.02.02 and v01.03.02 shows similar biases and dispersion (Lambert et al., 2019), as do comparisons from before and after the pixel spatial resolution change (Verhoelst et al., 2021). We extract good quality pixels based on the main quality flag (qa_flag) > 0.75, which removes retrievals flagged as bad and pixels over snow/ice or with cloud radiance fractions > 0.5, and resample them to our 15 km regional model (discussed later) using the bilinear interpolation. Since vertical column densities (VCDs) depend on assumed gas profile shape (i.e., they are quasi-observations), we recalculate those shape factors using profiles from our constrained chemical transport model. Shape factors are reestimated by calculating the ratio of the vertical column of total air to the simulated vertical column of NO_2_ multiplied by the mixing ratios of NO_2_ profile from the regional model (Martin et al., 2002).

Satellite remote sensing observations are usually far more stable than they are accurate. This can make the data practical for measuring relative changes in emissions but may necessitate the use of a bias correction for absolute emissions estimates. Moreover, the systematic and random errors associated with satellite retrievals may differ markedly from location to location. It is therefore crucial to thoroughly validate columns against independent observations. To this end, we compile statistics reported in several validation studies focusing on the TROPOMI tropospheric NO_2_ product and summarize their findings in Table 1. The most comprehensive global study to date is a comparison of TROPOMI tropospheric NO_2_ with that derived from 19 multi-axis differential optical absorption spectroscopy (MAX-DOAS) instruments (Verhoelst et al., 2021). This study indicates there is a low bias in TROPOMI tropospheric NO_2_ of −23 % to −37 % relative to MAX-DOAS at clean to moderately polluted sites and as large as −51 % at highly polluted sites. When considering all sites, the overall median bias in this study was found to be −37 %, with a dispersion of 3.5 × 10^15^ molec/cm^2^ (defined as half of the 68 % interpercentile). No obvious seasonal patterns were found in the biases. These results are consistent with other validation studies which have observed a low bias in TROPOMI tropospheric NO_2_ (Chan et al., 2020; Griffin et al., 2019; Judd et al., 2020). A potential significant source of bias in polluted regions is the relatively low spatial resolution (1 × 1°) TM5-MP prior profiles used in the TROPOMI air mass factor calculation. Several validation studies have shown the low bias in TROPOMI NO_2_ can be reduced in polluted regions by 5 %–17 % through the use of higher spatial resolution model a priori profiles or other improvements in the AMF calculation (Chan et al., 2020; Griffin et al., 2019; Judd et al., 2020; Zhao et al., 2020).

Directly incorporating these numbers into an inversion model is challenging, mainly because of spatiotemporal variability in the satellite errors. Ideally, the relationship between errors and retrieval inputs (e.g., albedo, scene radiance, profiles) would be used as an additional cost function in the inversion, commonly known as variational bias correction (e.g., Auligné et al., 2007). In the absence of such relationships, we use the biases reported in the validation studies.

In the case of NO_2_, we uniformly scale up the satellite tropospheric columns by 25 %. This bias estimate is derived by first assuming a 37 % low bias in the columns over polluted regions as reported by Verhoelst et al. (2021). In turn, this low bias can be mitigated somewhat by the application of high spatial resolution profiles in the air mass factor calculation, such as the ones used in this study. Table 1 summarizes the results from several TROPOMI validation studies at specific locations that calculated NO_2_ using model profiles with higher spatial resolution than the operational TROPOMI (1° × 1°) profiles (see Table 1 columns “modification” and “modified bias”). In these studies, modified columns show increases ranging from 0 %–5 %. Based on these results, we assume a low bias of 37 % can be mitigated by ~ 12 % through the use of high spatial resolution profiles, for a resulting total low bias of 25 %. This bias is likely not valid over pristine areas, where validation studies show lower biases in TROPOMI NO_2_ (Verhoelst et al., 2021; P. Wang et al., 2020; Zhao et al., 2020); nonetheless, we previously observed in Souri et al. (2020a) that the low signal-to-noise ratios of those column amounts resulted in small changes in the top-down emissions. We assume the errors of observations originate from two main sources: (i) the precision error provided with the data (*e*_precision_) and (ii) a fixed error estimated from comparisons to in situ measurements (*e*_const_). Mathematically, the final error is

(1) $$e_{O}^{2}=e_{\text{const }}^{2}+\frac{1}{n^{2}}\sum_{i=1}^{n}e_{\text{precision},\;i}^{2},$$

where *n* is the number of samples for a given grid and *e*_const_ is equal to 1.1 × 10^15^ molec/cm^2^ (< 6 × 10^15^ molec/cm^2^) in clean regions and 3.5 × 10^15^ molec/cm^2^ (> = 6 × 10^15^ molec/cm^2^) in moderately to highly polluted regions. These regions are defined based on the wide ranges reported in Verhoelst et al. (2021) (3–14 × 10^15^ molec/cm^2^ for moderately to highly polluted regions).

#### 2.1.2 MODIS AOD

To improve the simulation of total aerosol mass, we use the Collection 6 MODIS aerosol optical depth (AOD) from both Aqua (~ 13:30 LT) and Terra (~ 10:30 LT) platforms over both land and ocean (Levy et al., 2013) (available at https://ladsweb.modaps.eosdis.nasa.gov, last access: May 2020). We independently validate all three major products, namely the Deep Blue, the Dark Target, and combined Dark Blue products by comparing to AOD values measured by the Aerosol Robotic Network (AERONET) over Europe at the same time period as that of this study. Only good and very good (quality flag > = 2) pixels are selected for the comparison. The AERONET AOD data are computed based on the values at 500 nm and Ångström exponent in the 440–675 nm range. We collocate two datasets if they are within 10 km radius and less than 30 min apart. The Dark Blue product results in the best agreement (*r* > 0.87) with a high bias of < 0.05 (Figs. S3 and S4 in the Supplement). This product is therefore chosen for the data assimilation. We remove the bias and assign the value of the covariance matrix of observations to the RMSE values obtained from the comparison.

### 2.2 Surface measurements

UV photometry and chemiluminescence surface ozone and NO_2_ measurements all over continental Europe are used to investigate possible changes in their concentrations induced by the lockdown (https://discomap.eea.europa.eu/map/fme/AirQualityExport.htm, last access: June 2020). The NO_2_ chemiluminescence measurements are usually overestimated due to interferences from the NO_*z*_ family (PAN, organic nitrate, HNO_3_, etc.). We assume that the interferences are not significantly different between the baseline and lockdown mainly due to relatively low photochemistry in early spring (Lamsal et al., 2008) compared to summertime. Additionally, the correction needs a careful evaluation of the model with regards to the NO_*z*_ family whose measurements are not available in this case study.

More than 6450 meteorological stations archived on NOAA’s integrated surface database (https://www.ncei.noaa.gov/data/global-hourly/, last access: April 2020) are used to validate the performance of our weather model in terms of several prognostic inputs including ambient air temperature, air humidity, and *U* and *V* wind components.

### 2.3 WRF-CMAQ modeling

**Figure 1.**
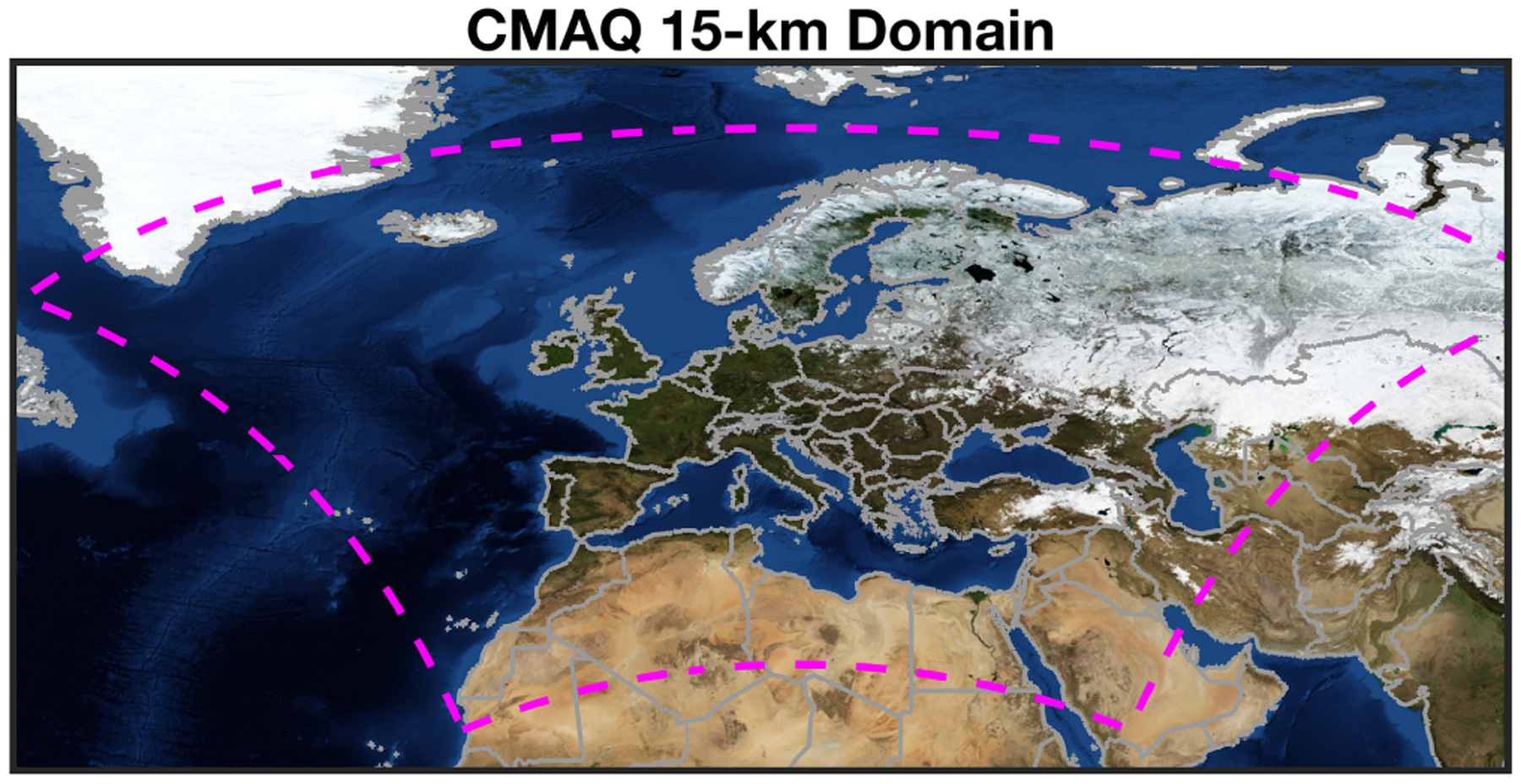
The WRF-CMAQ 15 km domain covering Europe. The background picture is based on the publicly available NASA Blue Marble (© NASA).

The regional air quality simulations at 15 × 15 km^2^ are carried out with the widely used CMAQ v5.2.1 (https://doi.org/10.5281/zenodo.1212601, US EPA Office of Research and Development, 2018) in conjunction with WRF v3.9.1 (Skamarock and Klemp, 2008) models. The models overlap and cover continental Europe and some portions of Africa and the Middle East. The domain consists of 483 east–west grids, 383 north–south grids, and 37 unevenly spaced eta levels (Fig. 1). The simulation time period is from March to May 2019 and 2020 (6 months). Since initial conditions (IC) and boundary conditions (BC) are taken from already spun-up National Centers for Environmental Prediction (NCEP) FNL (final) reanalysis and GEOS-Chem v12.9.3 (https://doi.org/10.5281/zenodo.3974569, The International GEOS-Chem User Community, 2020) runs, we only spin up the models for the month of February. The chemistry configuration of the CMAQ model mainly consists of CB05 with chlorine chemistry (gases) and AERO6 (aerosol). Hourly basis biogenic emissions are processed by the offline stand-alone Model of Emissions of Gases and Aerosols from Nature (MEGAN) v2.1 (Guenther et al., 2012) based on high-resolution plant functional maps made by Ke et al. (2012). The biogenic emission factors are estimated based on the PFT-specific information provided in Guenther et al. (2012). The biogenic VOCs include a wide range of compounds including isoprene, monoterpenes, aromatic VOCs, and methanol. Soil NO_*x*_ emissions are estimated by Yienger and Levy (1999). Lightning NO_*x*_ emissions are based on inline calculations involving convective precipitation rates and cloud vertical distributions. Lightning NO_*x*_ emissions are not constrained in the model. Anthropogenic emissions are based on the Community Emissions Data System (CEDS) inventory in 2014 (Hoesly et al., 2018). Diurnal scales are not considered for the anthropogenic emissions. We also output the CMAQ integrated process analysis quantifying the contribution of each process to the amount of compounds. The physical setting of WRF includes the Lin microphysics scheme (Lin et al., 1983), the Grell 3-D ensemble cumulus scheme (Grell and Dévényi, 2002), the Rapid Radiative Transfer Model for GCMs (RRTMG) radiation scheme, the Asymmetric Convective Model version 2 (ACM2) planetary boundary layer parametrization (Pleim, 2007), and the Pleim–Xiu land-surface scheme (Xiu and Pleim, 2001). To minimize the deviation of the model from the reanalysis data, we turn on the grid-nudging option with respect to wind, moisture, and temperature only outside of the planetary boundary layer (PBL) region. The inclusion of this option only outside of the PBL region is because we do not want the coarse reanalysis data to wash out the relatively high-resolution dynamics. Moreover, leaf area index and the sea surface temperature are updated every 6 h based on satellite measurements included in the reanalysis data. Extensive model evaluations based upon surface observations show a striking correspondence (Tables S1, S2 in the Supplement), which is indicative of reasonable energy budget and transport in our model.

### 2.4 Inverse modeling and data assimilation

To adjust the bottom-up emission inventories, we follow a non-linear joint inversion method proposed in Souri et al. (2020a). Briefly, a Gauss–Newton algorithm is utilized to incrementally solve Bayes’ quadratic function in analytical fashion. The posterior emissions are then derived by

(2) $$x_{i+1}=x_{a}+G\left[y-F\left(x_{i}\right)+K_{i}\left(x_{i}-x_{a}\right)\right],$$

where ***y*** is bias-corrected monthly averaged TROPOMI NO_2_ and HCHO observations (see Sect. S1), ***x***_*a*_ (or ***x***_0_) is the prior emissions, ***x***_*i*_ is the posterior emission at the *i*th increment, *F* is the forward model (here WRF-CMAQ) to project the emissions onto the columns’ space, **G** is the Kalman gain,

(3) $$G=S_{\text{e}}K_{i}^{T}{\left(K_{i}S_{\text{e}}K_{i}^{T}+S_{\text{o}}\right)}^{-1},$$

and **K**_*i*_ (= **K**(***x***_*i*_)) is the Jacobian matrix calculated explicitly from the model using the finite difference method by perturbing separately NO_*x*_ and VOC emissions by 20 %. The perturbations are applied for each iteration. The model outputs along with Jacobians and emissions are spatiotemporally co-registered with the observations. **S**_o_ and **S**_e_ are the error covariance matrices of the observations and emissions. Similar to Souri et al. (2020a), the prior errors in anthropogenic NO_*x*_ and VOCs emissions are set to 50 % and 150 %, respectively. In terms of the biogenic emissions, the errors are set to 200 % for both NO_*x*_ and VOCs. The instrument covariance matrices are populated with the squared sum of the aforementioned errors based on the compilation of the validation studies and precision errors provided with the data (Eq. 1). Both error matrices are assumed diagonal. The inversion window is monthly meaning we have three separate correction factors in months of March, April, and May. The covariance matrix of the a posteriori is calculated by

(4) $${\hat{S}}_{\text{e}}=(I-G\hat{K})S_{\text{e}},$$

where $\hat{K}$ is the Jacobian from the *i*th iteration. Here, we iterate Eq. (2) three times. The averaging kernels (**A**) are given by

(5) $$A=I-{\hat{S}}_{\text{e}}S_{\text{e}}^{-1}.$$

Not only does this method considers non-linear chemical feedback among NO_2_–HCHO–NO_*x*_–VOC by simultaneously incorporating the HCHO and NO_2_ in the inversion framework, it also permits quantification of **A** that explicitly explains the amount of information obtained from the observation. Low **A** indicates low **G**, making the a posteriori rather independent of the observational constraint.

An important caveat with this inversion system is that we do not take the model parameter error (such as errors in chemistry, cloud microphysics, and PBL) into account. To properly estimate the forward model parameter errors, one needs to calculate the sensitivity matrix of the columns to the model parameters combined with the sensitivity matrix of the columns to the emissions (**K**) (Rodgers, 2000). The former calculation is computationally expensive. Moreover, the spatiotemporal varying model parameter errors may not be known in detail. The consequence of disregarding the model parameter errors is an overconfidence in the top-down estimates (i.e., an overestimation of AKs).

We also correct total aerosol mass by daily assimilating the MODIS Dark Blue AOD observations following the algorithm discussed in J. Jung et al. (2019). Briefly, the assimilation framework uses a modified optimal interpolation method adjusting uniformly all relevant aerosol masses in a column as a function of a weighted-distance and appropriate errors.

## 3 Results and discussion

### 3.1 Variability of NO_2_ columns seen by TROPOMI

**Figure 2.**
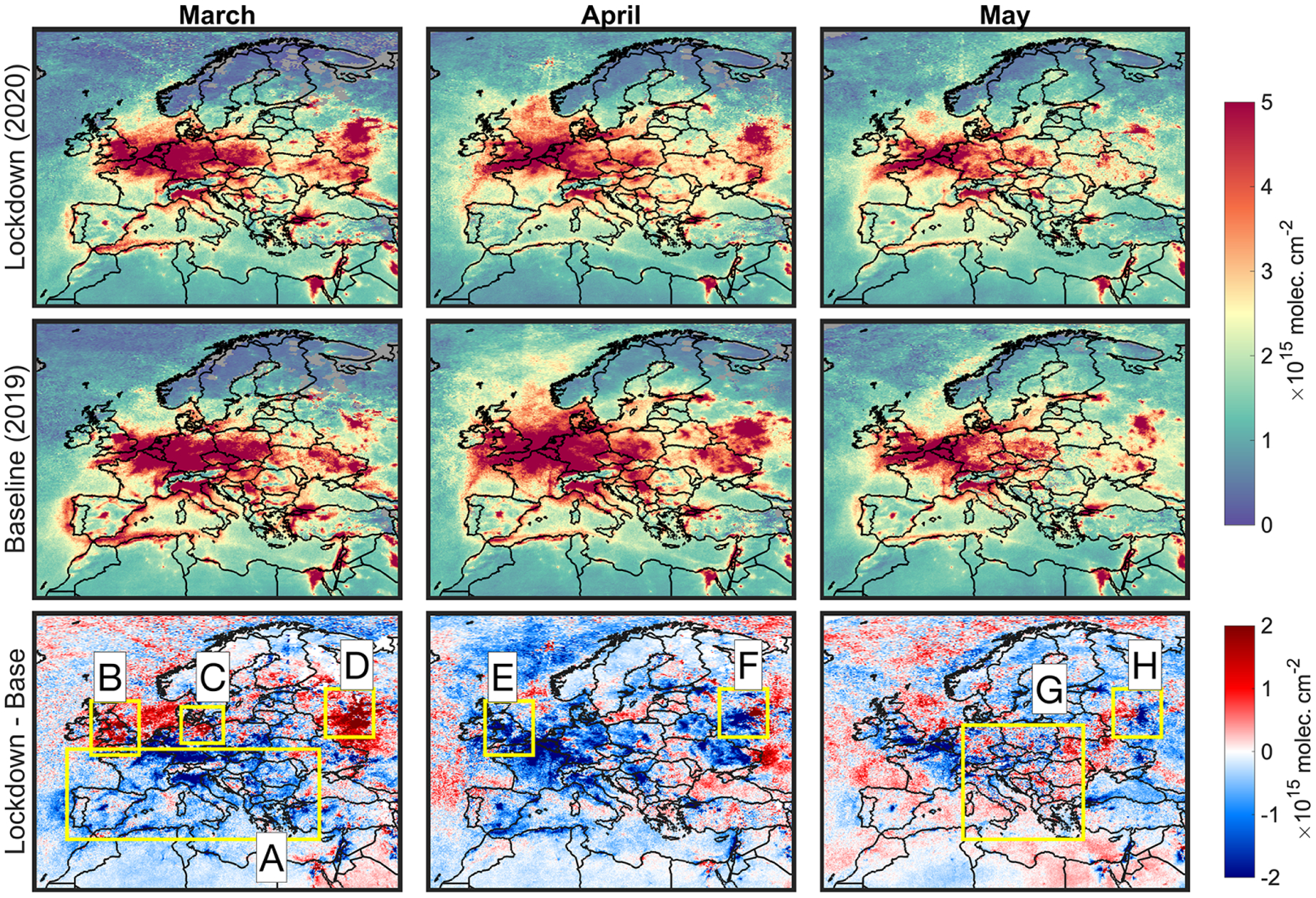
First row: maps of tropospheric NO_2_ from the TROPOMI sensor during months of March, April, and May in 2020 (lockdown). Second row: same as the first row but for the baseline year (2019). Last row: difference of the columns in 2020 with respect to those of 2019. All columns are corrected for the bias and their AMFs are recalculated iteratively based on the posterior profiles derived from our inverse modeling practice. The satellite-derived columns are subject to errors, so a direct interpretation of their magnitudes cannot be performed in a robust manner.

We assess difference maps of NO_2_ columns (and HCHO in Sect. S1) in 2020 with respect to those in 2019 during the months of March, April, and May. The difference maps along with the absolute values of the tropospheric NO_2_ columns are shown in Fig. 2. Regardless of the year, we observe a noticeable reduction in NO_2_ as we approach warmer months which can be explained by increases in OH concentrations (higher water vapor content, solar radiation, and O_3_ levels), faster vertical mixing due to larger sensible fluxes (more diluted columns for a given receptor due having a greater chance of experiencing stronger winds in higher altitudes), and a reduction in temperature-dependent light-duty diesel NO_*x*_ emissions (Grange et al., 2019). This sequential decline of NO_2_ obscures the quantitative interpretation of the satellite observations in two ways: first, as noted by Silvern et al. (2019), the free tropospheric background NO_2_ levels, which are highly uncertain, becomes comparable to those located in near-surface areas, and second, the relatively lower signal-to-noise ratios reduce the amount of information that we can obtain for inverting NO_*x*_ emissions (discussed later).

The anomaly map (2020 vs. 2019) in March indicates pronounced decreases in tropospheric NO_2_ columns over several countries including France, Spain, Italy, and Germany (box A). In contrast, we see increases in the magnitude of the NO_2_ columns over some portions of the UK excluding London (box B), northeastern Germany (box C), and Moscow, Russia (box D). A recent study (Barré et al., 2021) observed roughly the same tendency which was attributable to meteorological changes. While those changes are indeed an important piece of information, we should recognize that the degree of the enforced restrictions varies temporally; moreover, changes in emission heavily rely on the dominant emission sector (e.g., mobile or industry). For instance, according to TASS press (https://tass.com/society/1144123, last access: September 2020), Russian governments did not take significant measures to control the virus before 15 April, immediately evident in the large NO_2_ enhancement over Moscow in March (box D). During the next two months (April and May), we observe a major turnaround over this city (boxes F and H). In May, the anomaly of the tropospheric NO_2_ suggests that the reduction in NO_*x*_ emissions abruptly experiences a hiatus in central Europe (box G). However, it is crucial to note that these maps are based upon sporadic clear-sky pixels that might obscure the full portrayal of emissions changes happening throughout the period (discussed later).

### 3.2 Top-down estimates of NO_*x*_ emissions

Following the inversion and the data assimilation frameworks, we adjust the total amounts of VOC, NO_*x*_ emissions, and aerosols mass using TROPOMI HCHO, NO_2_, and MODIS AOD observations. We focus on the topic of gas-phase chemistry (i.e., ozone and its precursors) implying that the aerosol data assimilation is carried out to partially remove errors associated with radiation (e.g., J. Jung et al., 2019) or heterogenous chemistry (Jacob, 2000); therefore, the aspect of aerosol changes induced by the lockdown will be examined in a separate study. Furthermore, we observe a relatively weak observational constraint from TROPOMI HCHO on VOC emissions, especially in higher latitudes; accordingly, the relevant discussion on this subject is presented in Sect. S2.

**Figure 3.**
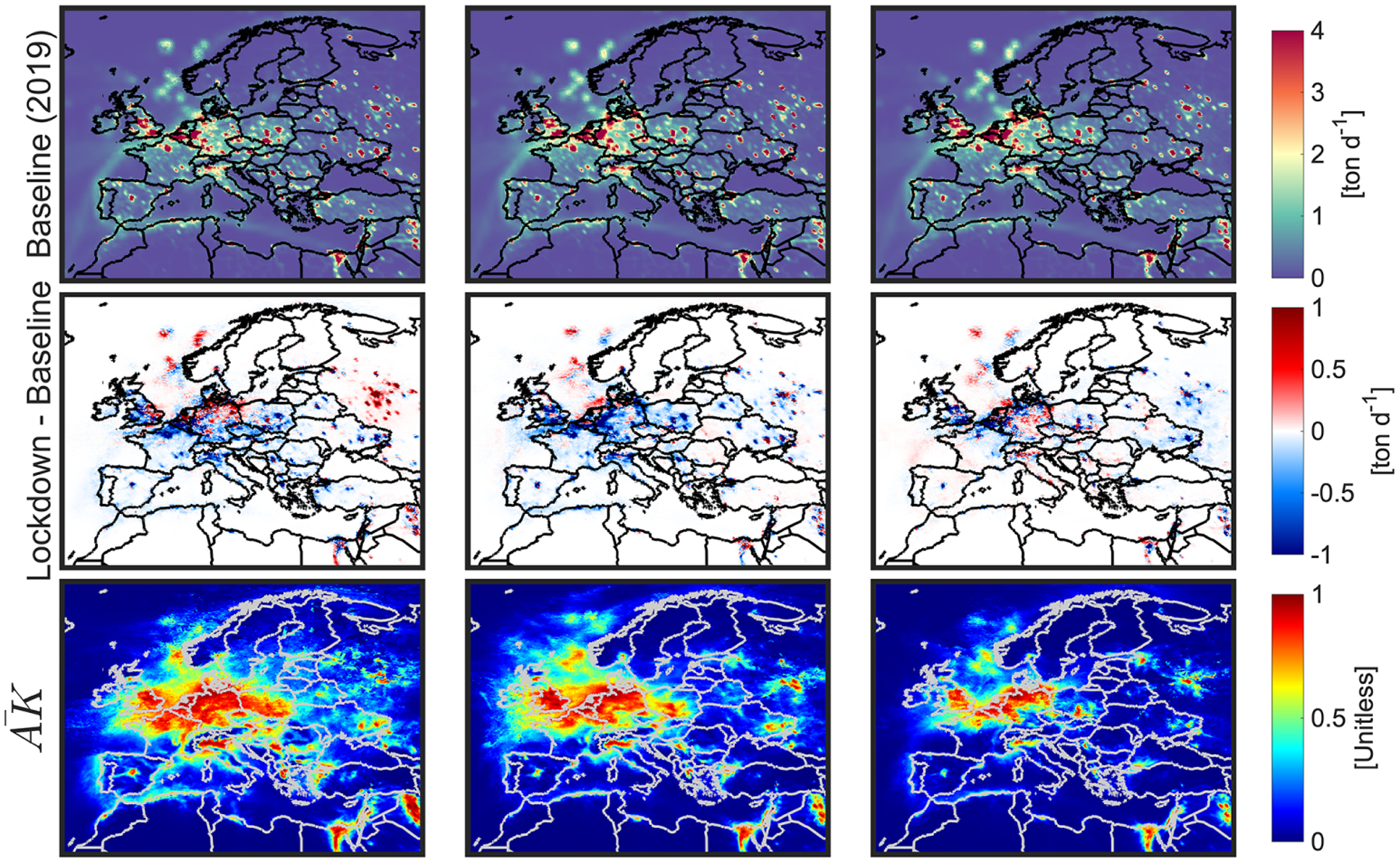
Top-down estimates of total NO_*x*_ during months of March, April, and May in 2019 (baseline) and the differences between emission in 2020 (lockdown) and 2019. To infer the magnitude of emissions in 2020, the second row should be added to the first one. Both TROPOMI HCHO and NO_2_ observations are jointly used to estimate these numbers. Averaging kernels (mean values based on both 2019 and 2020 estimates) describe the level of credibility of the estimate which is heavily dependent on the TROPOMI signal-to-noise ratios.

The spatial distributions of magnitude of the top-down NO_*x*_ and their corresponding changes and averaging kernels are shown in Fig. 3. Moreover, the monthly values of the a posteriori and the a priori are shown in Figs. S5 and S6. It is worth emphasizing that we use identical prior values in terms of anthropogenic emissions in both years.

According to Fig. 3, large averaging kernels associated with NO_*x*_ emissions are confined in high-emitting regions suggesting that the most valid estimates can be found in areas undergoing strong TROPOMI NO_2_ signals. We observe an improvement in the statistics associated with simulated surface NO_2_ using the posterior emissions compared to the surface measurements in many places around Europe, with an exception in northeastern Germany where TROPOMI NO_2_ observations deviates the model from the measurements (Figs. S9–S12; Tables S3, S4). The large underestimation of the model in terms of surface NO_2_ concentrations is most likely due to the underestimation of the CEDS inventory (e.g., Fig. 12 in Sun et al., 2021). However, it is worth noting that the disagreement between the model and the surface measurements does not solely reflect the uncertainty in the emissions. A major complication arises from the fact that the point measurements represent concentrations locally, whereas the model grids (15 × 15 km^2^) are (at best) the average of infinitesimal points integrated over the grid space. Essentially, no one should expect that these quantities will completely line up, unless one transforms the point measurements to the grids (i.e., rasterization) by carefully modeling the spatial auto-correlation (or semivariograms) of the point data (Souri et al., 2021b). Additionally, there is uncertainty about the chemical mechanism utilized in the model. In particular, Souri et al. (2017) observed a large overestimation (~ factor of 4) of daily averaged total nitrate $\left({\text{HNO}}_{3}+{\text{NO}}_{3}^{-}\right)$ in the CB05/AERO6 mechanism despite moderately reasonable nitrate $\left({\text{NO}}_{3}^{-}\right)$ simulations. This was attributed to a large overestimation of N_2_O_5_ hydrolysis rate (Bertram and Thornton, 2009) which is the primary loss pathway of NO_*x*_ in low photochemically active regions (Shah et al., 2020). The interferences from the NO_*z*_ family on the surface measurements might be still present in springtime in midlatitudes (~10 %–30 %) (Lamsal et al., 2008). Last but not least, the PBL parameterization controlling the level of vertical mixing rates has errors primarily due to soil moisture not being observationally constrained in the model (Huang et al., 2021).

The discrepancies between the simulated tropospheric NO_2_ columns versus TROPOMI are mitigated by the inversion (Figs. S13 and S14). Immediately apparent in Fig. 3 is a strong correlation between anomaly maps of TROPOMI tropospheric NO_2_ (Fig. 2) and those of top-down emissions. We observe reductions in NO_*x*_ emissions in March (14 %–31 %) in several major cities including Paris, London, Madrid, and Milan; the reductions further expand to Rome, Brussels, Frankfurt, Warsaw, Kyiv, Moscow, and Belgrade with higher magnitudes (34 %–51 %) in April. In general, the level of NO_*x*_ reduction is somewhat higher in April relative to months of March and May possibly due to temporal variabilities associated with the restrictions; for example, the UK and Poland governments enforced the restrictions starting in the last week of March to the middle of April (see Fig. S1 in Okruszek et al., 2020; https://www.bbc.com/news/uk-51981653, last access: March 2020). The decreased anthropogenic NO_*x*_ emissions in the Strait of Gibraltar and Alboran Sea reveal reportedly reduced ship activities (United Nations Conference on Trade and Development Report, 2020). The numbers in May indicate that several countries in central and eastern Europe (shown in box G in Fig. 2) likely eased coronavirus lockdown restrictions, a picture that has yet to be verified by surface measurements (discussed later).

### 3.3 Disparities in near-surface concentrations suggested by the constrained model versus those by in situ measurements

#### 3.3.1 NO_2_

It is necessary to examine whether the constrained model can precisely represent the changes observed by surface measurements. Several factors can complicate this analysis: (i) having overconfidence in the constrained model where the satellite observations used were uncertain; this problem can be addressed by considering grid cells whose averaging kernels are above a threshold (here 0.5), (ii) not accounting for spatial representativity function when it comes to directly comparing two datasets at different scales (i.e., point measurements vs. the model grids); a statistical construction of the spatial representativity function (Janjić et al., 2018; Souri et al., 2021b) requires a dense observational network so that we can build a semivariogram; instead, we only consider model grid cells having more than two stations; those observations then are then averaged, (iii) interferences of the NO_*z*_ family on NO_2_ chemiluminescence measurements (Dickerson et al., 2019) which can be partly discounted when calculating differences, (iv) model uncertainties, especially with respect to turbulent and convective fluxes that are heavily determined by representing local heterogeneity of forces and non-hydrostatic dynamics (Emanuel, 1994), all of which are challenging to fully resolve in a 15 km resolution.

**Figure 4.**
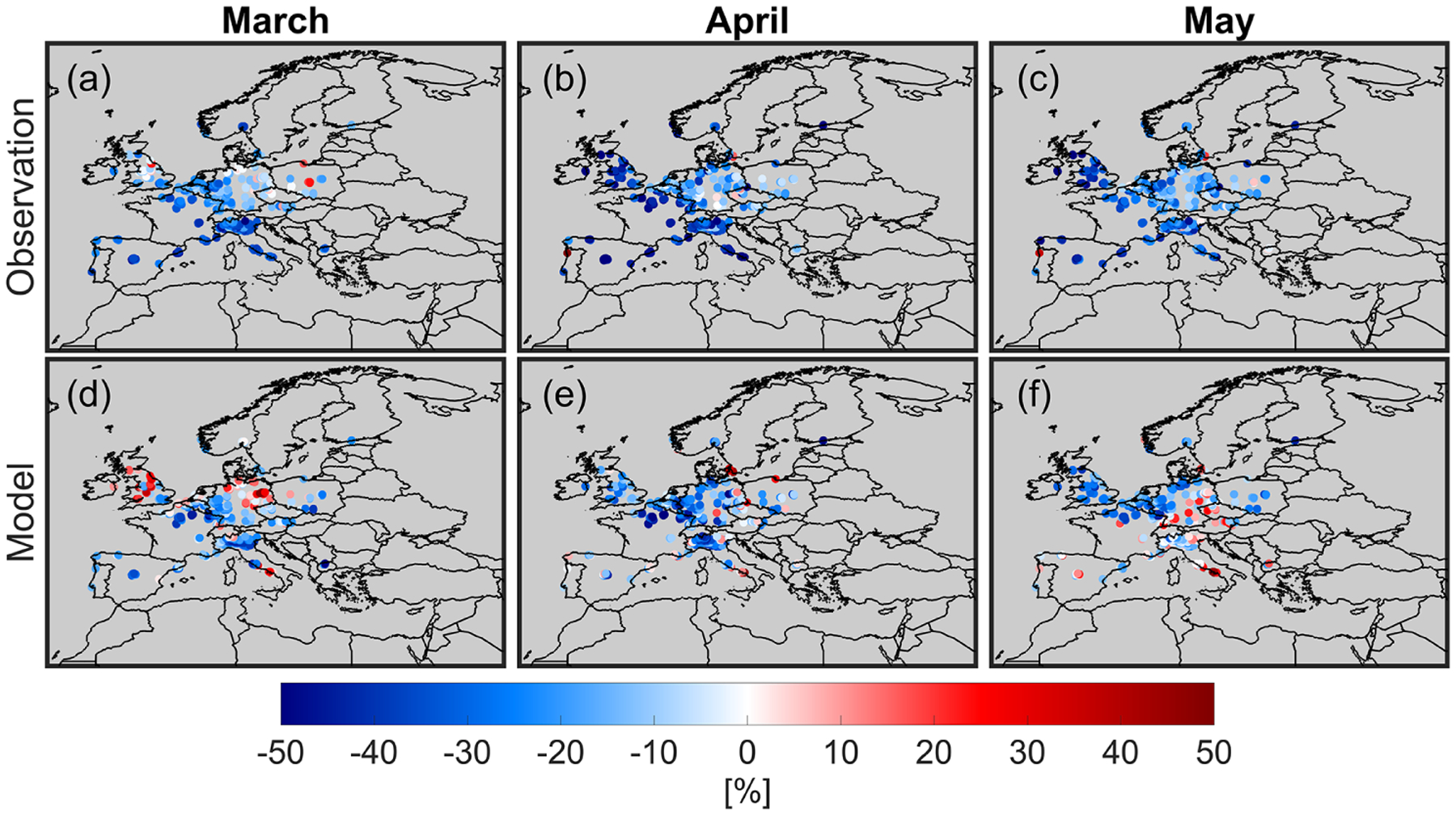
Scatter maps of relative changes in surface NO_2_ concentrations suggested by the European air quality network (**a**, **b**, **c**), and the constrained model (**d**, **e**, **f**). Results are daily averaged. We only consider grid cells whose averaging kernels (from the NO_*x*_ inversion) are above 0.5. Furthermore, grid cells having more than two stations are only included to partly account for the spatial representativity factor. Surface concentrations are not accounted for the NO_*z*_ family interferences.

**Figure 5.**
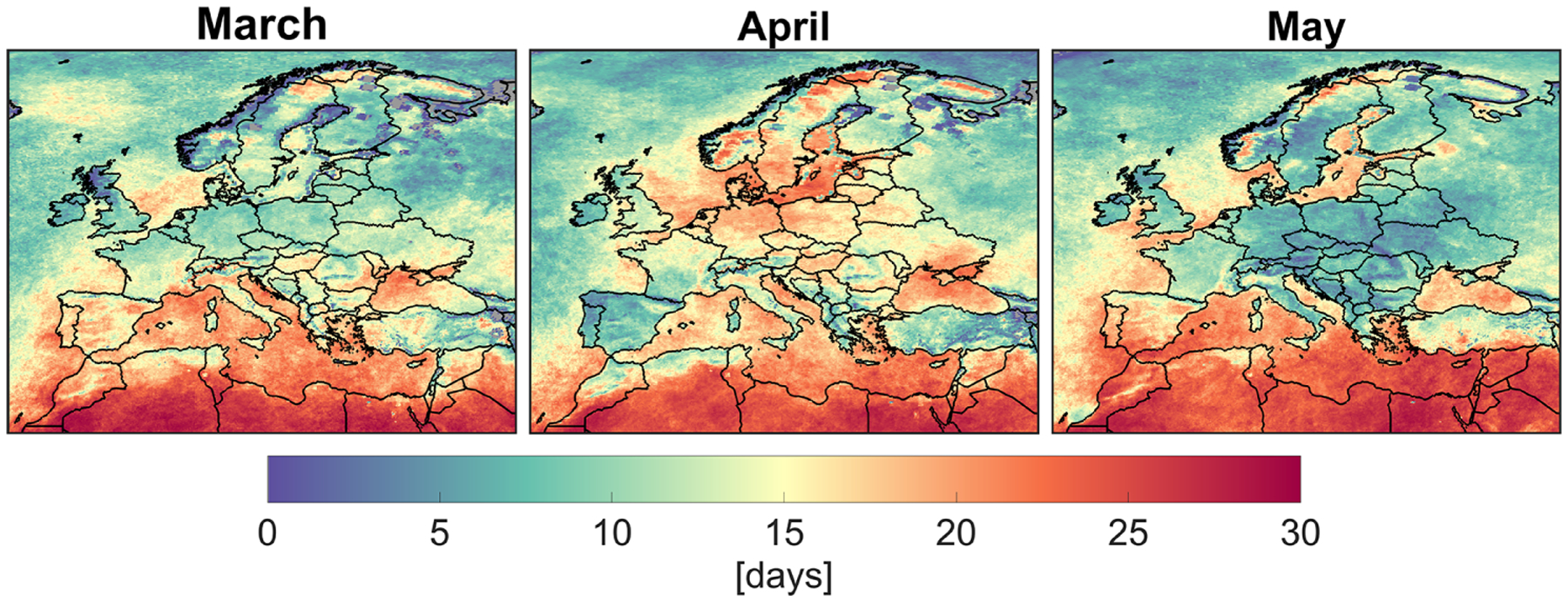
The average number of good quality (qa_flag > 0.75) TROPOMI tropospheric NO_2_ days observed at 15 × 15 km^2^ in 2019 and 2020. These numbers are heavily affected by cloudiness.

With these caveats in mind, we plot the daily averaged changes of surface NO_2_ concentrations in 2020 relative to 2019 derived by the model and the European air quality network for the months of March, April, and May (Fig. 4). Large gaps in Fig. 4 are caused by considering grid cells with averaging kernels > 0.5 and number of samples > 2. The constrained model correlates reasonably well with the changes observed by the surface measurements in April, but it fails to fully reflect those in March and May. The surface measurements in March reinforce increases (or negligible changes) in NO_2_ in northeastern Germany and the UK, although the magnitudes are not as large as those suggested by the model (and TROPOMI NO_2_ columns). A number of factors can contribute to these large discrepancies: (i) the surface measurements are present throughout the month of March, whereas TROPOMI data are frequently absent due to cloudiness resulting in some degree of temporal representativity issues; (ii) the statistics used for the TROPOMI bias correction may not always hold true, since each individual pixel can deviate from the norm of the reported biases; (iii) the shape of NO_2_ profiles simulated by the WRF-CMAQ can sometimes be uncertain due to errors in the PBL parameterization or the difficulties with resolving the non-hydrostatic components (where vertical motions are comparable to horizontal ones) (e.g., Pouyaei et al., 2021); this complication can result in unrealistic changes in the columns. The constrained model tends to consistently underrepresent the decline in NO_2_ in March (model: −11 ± 21 %, observation: −19 ± 16 %), April (model: −21 ± 17 %, observation: − 29 ± 21 %), and May (model: −12 ± 18 %, observation: − 25 ± 20 %). The frequency of TROPOMI data heavily impacted by cloudiness is an important factor effectively leading to the underrepresentation of the model in the course of a month. Figure 5 depicts the average number of days that TROPOMI was able to sample in both years (individual years are shown in Figs. S18 and S19). There is a strong degree of correlation between the frequency of the data and the discrepancy between the model and the surface observations. This is especially the case for May when we see too few days to be able to realistically reproduce NO_2_ changes.

Given the reasonable performance of our model at reproducing the changes observed over the surface in April, a result of abundant samples from TROPOMI, we only focus on this month for the subsequent analysis.

#### 3.3.2 Ozone

**Figure 6.**
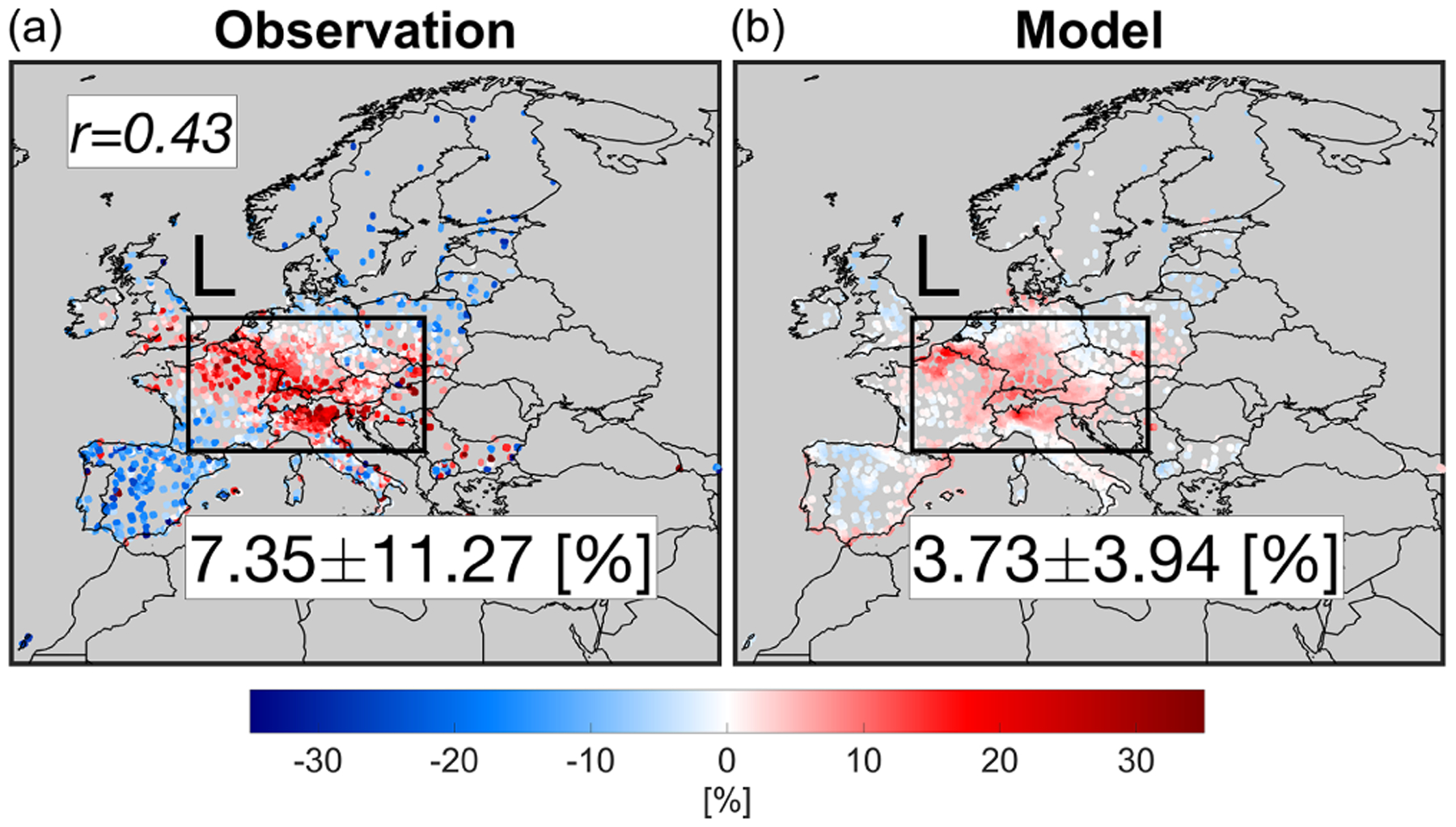
Changes in surface MDA8 ozone concentrations suggested by the observation (**a**) and the constrained model (**b**) in April 2020 relative to those in 2019. The numbers are based on the box L region.

Figure 6 depicts the changes in maximum daily 8 h average (MDA8) surface ozone concentrations suggested by the measurements and the constrained model in April 2020 with respect to 2019. Immediately obvious from the observations is the elevated surface ozone concentrations up to 32 % in places where NO_*x*_ emissions drastically decreased such as Germany, Italy, France, UK, Switzerland, and Belgium (shown as box L). This tendency, potentially driven by ozone chemistry (Sicard et al., 2020; Shi and Brasseur, 2020; Gaubert et al., 2021; Salma et al., 2020; Lee et al., 2020) and/or meteorology (Lee et al., 2020; Wyche et al., 2021; Ordóñez et al., 2020), has drawn much attention. The challenge is to set up a model that is characteristic of such a complex tendency (e.g., Parrish et al., 2014). Encouragingly, our constrained model does have skill in describing the ozone enhancements over the whole domain (*r* = 0.43). In the proximity of central Europe (shown as box L), the enhanced MDA8 ozone concentration observed by the observations is 7.35 ± 11.27 % (+3.76 ppbv), which is nearly a factor of 2 larger than that of the model (3.73 ± 3.94 %, +1.79 ppbv).

**Figure 7.**
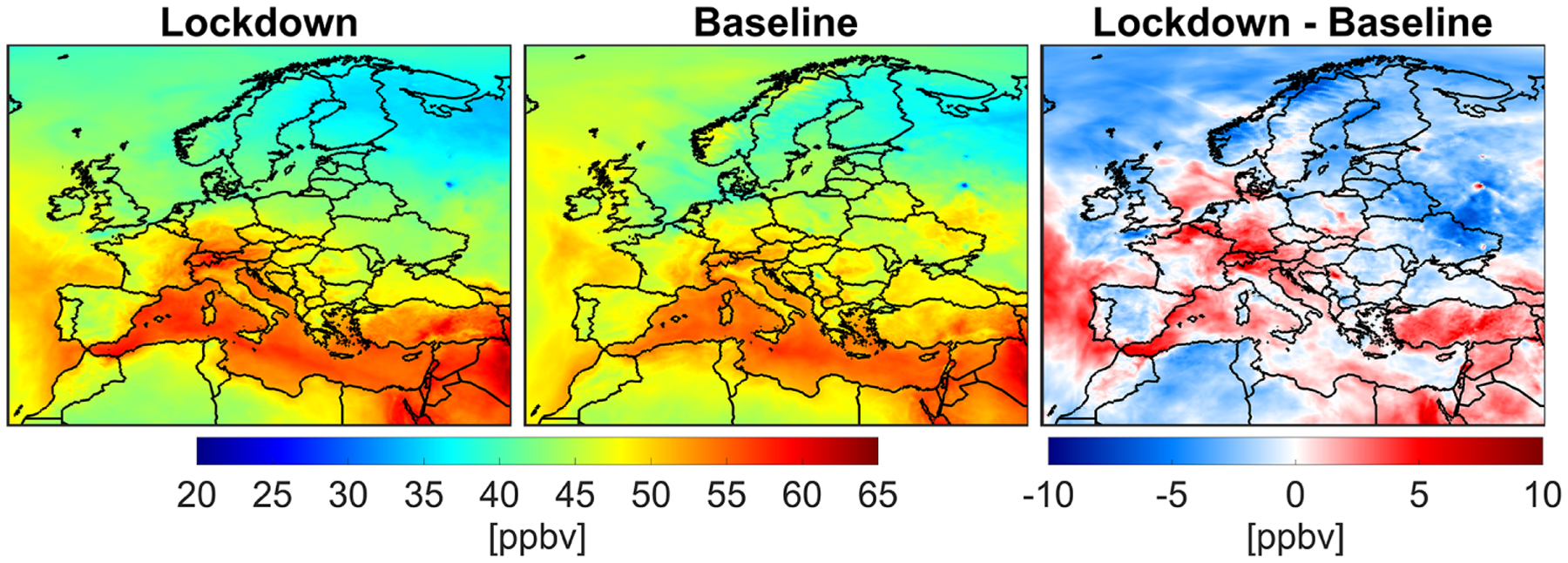
Simulated surface MDA8 ozone concentration using the constrained model in the month of April 2020 (lockdown), April 2019 (baseline), and their difference.

We plot the simulated MDA8 surface ozone concentrations in April 2020 (lockdown), April 2019 (baseline), and their differences in Fig. 7. Surface ozone concentrations show a strong latitudinal gradient with lower values in higher latitudes, underscoring the importance role of solar radiation in the formation of ozone. Meanwhile, the Mediterranean basin is prone to elevated concentrations of ozone resulting from different factors including calm weather, the transport from neighboring countries, atmospheric recirculation in coastal environments, and local emissions (Lelieveld et al., 2002). While we observe a strong variability in the difference map, signaling various sources and sinks (discussed later), three distinctive features in 2020 in comparison to 2019 are evident: (i) higher concentrations over the central Europe (up to 5 ppbv), (ii) lower concentrations in eastern Europe (−2.67 ± 1.65 ppbv) due to the 2019 biomass burning activities (see Sects. S1 and S2) and larger snow cover fraction accelerating photolysis (e.g., Rappenglück et al., 2014), and (iii) lower values on the Iberian Peninsula (−0.51 ± 1.41 ppbv) (Ordóñez et al., 2020).

**Figure 8.**
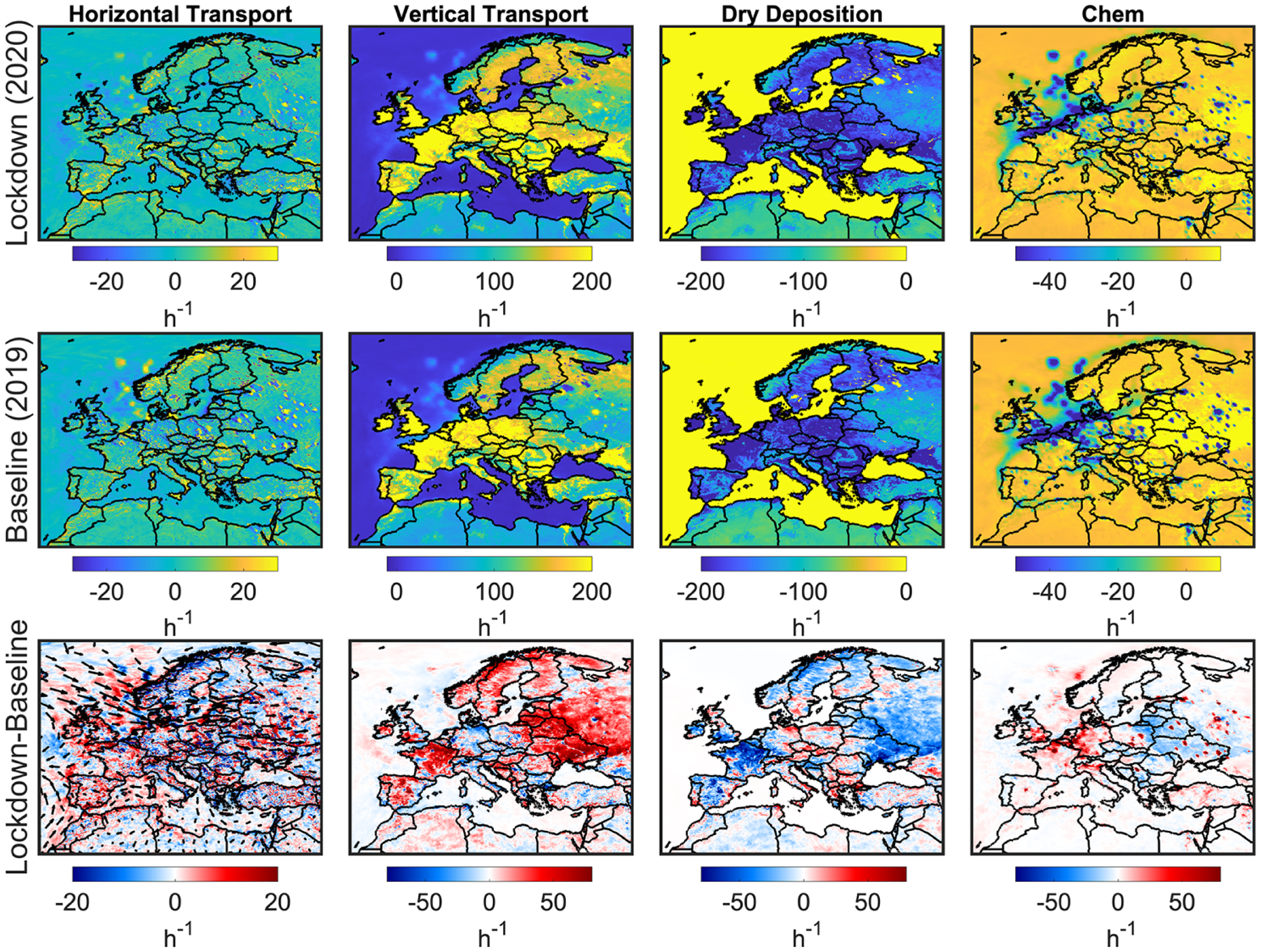
Surface process tendencies (h^−1^) including horizontal transport (advection plus diffusion), vertical transport (advection plus diffusion), dry deposition, and chemistry. Positive (negative) values mean source (sink) of ozone. These outputs are based on the constrained model. Wind vectors are the difference.

While the remaining model uncertainty could be either improved or characterized by including more observations (if available), reconfiguring the physiochemical mechanisms used, and constraining chemical boundary conditions, it is imperative to gauge the contribution of each process (i.e., transport, chemistry, etc.) in forming ozone changes. Here, we mainly make use of the CMAQ process analysis. A direct use of the process analysis output (in units of ppbv/h) can be confusing as both physiochemical processes and underlying concentrations are inextricably linked together. To be able to isolate each process (in units of h^−1^), we normalize the outputs by ozone concentrations. We average each process at the same hours used in calculating MDA8. Figure 8 shows the major model processes, namely horizontal transport (horizontal advection plus diffusion), vertical transport (vertical advection plus diffusion), dry deposition, and chemistry in 2020, 2019, and their differences. Positive (negative) values indicate a source (sink) for ozone. Regarding the horizontal transport, the values mostly follow the transport pattern and are dependent on whether the advected air mass is more or less polluted. The vertical transport correlates with the planetary boundary layer height (PBLH) which is an indicator of the atmospheric stability and turbulence, although we should not rule out the impact of the subgrid convective transport that can occur sporadically. Low PBLHs are usually associated with more stable (or sometimes capping inversion) and weaker vertical mixing (e.g., Nevius and Evans, 2018). Vertical transport which is majorly dictated by the vertical diffusion is by far the most influential factor in the magnitude of ozone (e.g., Cuchiara et al., 2014). In contrast to NO_2_ and HCHO, a stronger vertical diffusion increases surface ozone due to positive gradients of ozone with respect to altitude. However, the aerodynamic resistance controlling dry deposition velocity (Seinfield and Pandis, 2006) is also a function of turbulent transport. For example, during daytime, intensified turbulence exposes more pollution to surface deposition. It is for this reason that we see the dry deposition process largely counteracting vertical transport. This will leave the chemistry process as the major driver of the ozone changes.

We separately sum the quantities of the physical processes and PO_3_ contributing to MDA8 surface ozone changes binned to box L. The physical processes lead to −4.83 ppbv changes in the MDA8 ozone mainly due to a relatively larger dry deposition in 2020, whereas P(O_3_) contributes to +5.89 ppbv. The net effect is +1.06 ppbv, which is slightly smaller than the simulated changes in MDA8 ozone in this region (+1.79 ppbv). This apparent discrepancy is caused by the differences in boundary and initial conditions which are not quantifiable by the process analysis and would require additional sensitivity tests. Nonetheless, we believe these numbers should provide convincing evidence of the fact that chemistry has promoted the enhancements of surface ozone during the lockdown.

**Figure 9.**
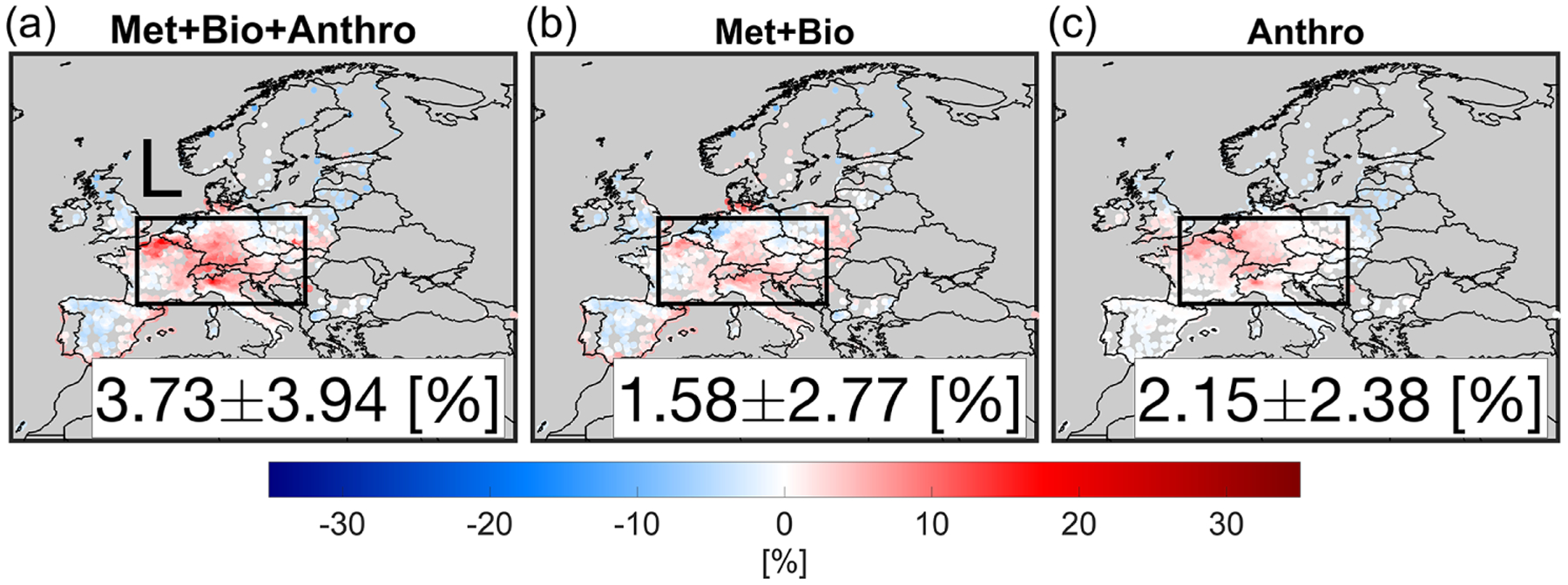
Simulated MDA8 surface ozone difference between April 2020 with respect to April 2019 including (**a**) dynamical meteorology, biogenic and anthropogenic emissions, (**b**) dynamical meteorology and biogenic emissions, and (**c**) the subtraction of the previous scenarios isolating dynamical anthropogenic emissions. Emissions used for these experiments are based on the top-down estimates.

Chemistry is also a function of meteorology, specifically solar radiation and temperature. A typical scenario to isolate emissions from meteorology is by running the model with fixed anthropogenic emissions (and boundary conditions) and subtracting the outputs from the variable emission output. Figure 9 shows the contribution of anthropogenic emissions (VOCs and NO_*x*_) to the changes seen over the surface. The anthropogenic emissions make up roughly 58 % of the changes. The map is strongly in line with the changes in NO_*x*_ emissions constrained by TROPOMI. The impact of meteorology plus biogenic changes (the former is dominant) highly correlates with anomalies in both surface air temperature and photolysis rates dictated by synoptic conditions (Fig. S17). We observe negligible ozone changes due to emissions over the Iberian Peninsula, reinforcing the significance of the meteorological impacts (Ordóñez et al., 2020).

### 3.4 Ozone chemistry

**Figure 10.**
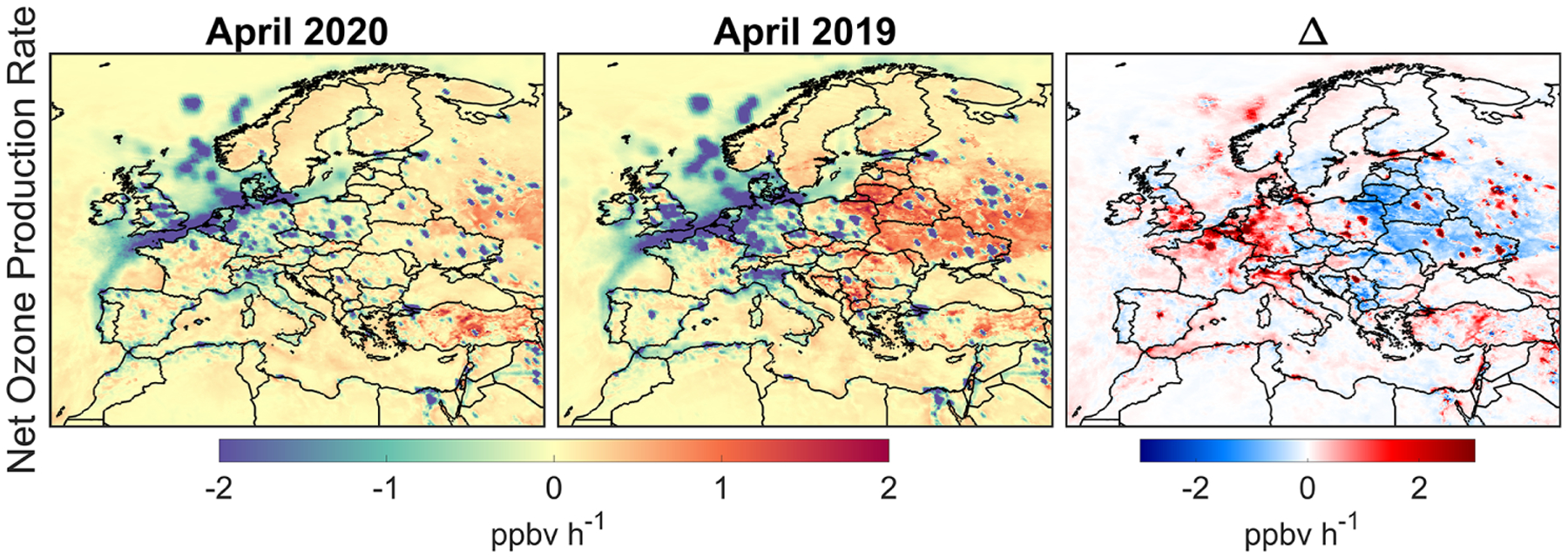
Numerically solved net ozone production rates based on the WRF-CMAQ simulations using the constrained emissions by the satellite data in April 2020, 2019, and the difference. These values are over the surface and are averaged during the MDA8 hours.

Figure 10 shows the numerically solved ozone production rates (PO_3_) simulated by the constrained model during the MDA8 hours period. We observe positive PO_3_ in less polluted areas and eastern Europe where biomass burning activities occurred in 2019 (see Sects. S1 and S2), while negative PO_3_ in major cities. Negative values in PO_3_ are indicative of either loss in O_3_ or O_3_–NO–NO_2_ partitioning. The difference in PO_3_ between the two years suggests that the ozone enhancement in box L is caused by a reduction in negative PO_3_ in 2020 over major cities compared to 2019. To examine which pathways are contributing to this pattern, we attempt to analytically reproduce the numerically solved PO_3_ (Fig. 10) through two different equations: the first equation, widely applied in photochemically active environments, is as follows (Kleinman et al., 2002):

(6) $$\begin{array}{l} \text{P}\left({\text{O}}_{3}\right)=k_{{\text{HO}}_{2}+\text{NO}}\left[{\text{HO}}_{2}\right][\text{NO}]+\sum k_{{\text{RO}}_{2i}+\text{NO}}\left[{\text{RO}}_{2i}\right][\text{NO}]\\ -k_{\text{OH}+{\text{NO}}_{2}+M}[\text{OH}]\left[{\text{NO}}_{2}\right][M]\\ -k_{{\text{HO}}_{2}+{\text{O}}_{3}}\left[{\text{HO}}_{2}\right]\left[{\text{O}}_{3}\right]-k_{\text{OH}+{\text{O}}_{3}}[\text{OH}]\left[{\text{O}}_{3}\right]\\ -k_{\text{O}{(}^{1}\text{D})+{\text{H}}_{2}\text{O}}\left[\text{O}{(}^{1}\text{D})\right]\left[{\text{H}}_{2}\text{O}\right]\\ -L\left({\text{O}}_{3}+\text{VOCs}\right). \end{array}$$

This equation yields negative values only if the O_3_ loss pathways including NO_2_ + OH, HO_*x*_ + O_3_, O^1^D + H_2_O, and O_3_ + VOCs dominate over the first two terms. The second equation, which is independent of RO_2_ and HO_2_ concentrations (Thornton et al., 2002), is

(7) $$\text{P}\left({\text{O}}_{3}\right)=j{\text{NO}}_{2}\left[{\text{NO}}_{2}\right]-k_{\text{NO}+{\text{O}}_{3}}\left[{\text{O}}_{3}\right][\text{NO}].$$

In summer, this equation tends to be positive during early afternoon, almost zero during afternoon (steady state), and negative in early morning (or night) in which the second term (O_3_ titration) is leading. Any abrupt changes in NO_*x*_ and VOC, and photolysis can directly affect Eq. (7) moving PO_3_ out of the diel steady state. The assumption of the steady state (PO_3_ from Eq. 7 is equal to zero) is also not valid if an air parcel is in the vicinity of high-emitting NO_*x*_ sources (Thornton et al., 2002).

**Figure 11.**
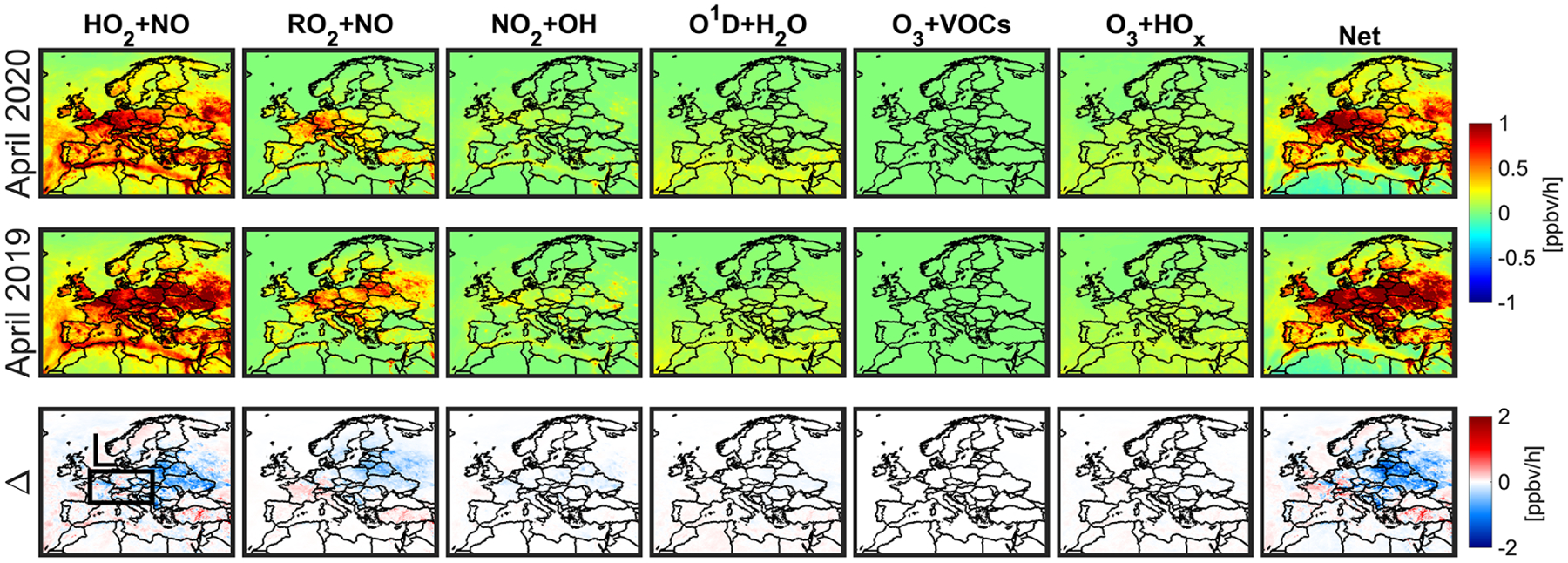
Surface chemical processes involved in Eq. (6) (ppbv/h) pertaining to the production and loss of ozone in April 2020 (lockdown) and 2019 (baseline) during MDA8 hours. These outputs are based on the constrained model.

**Figure 12.**
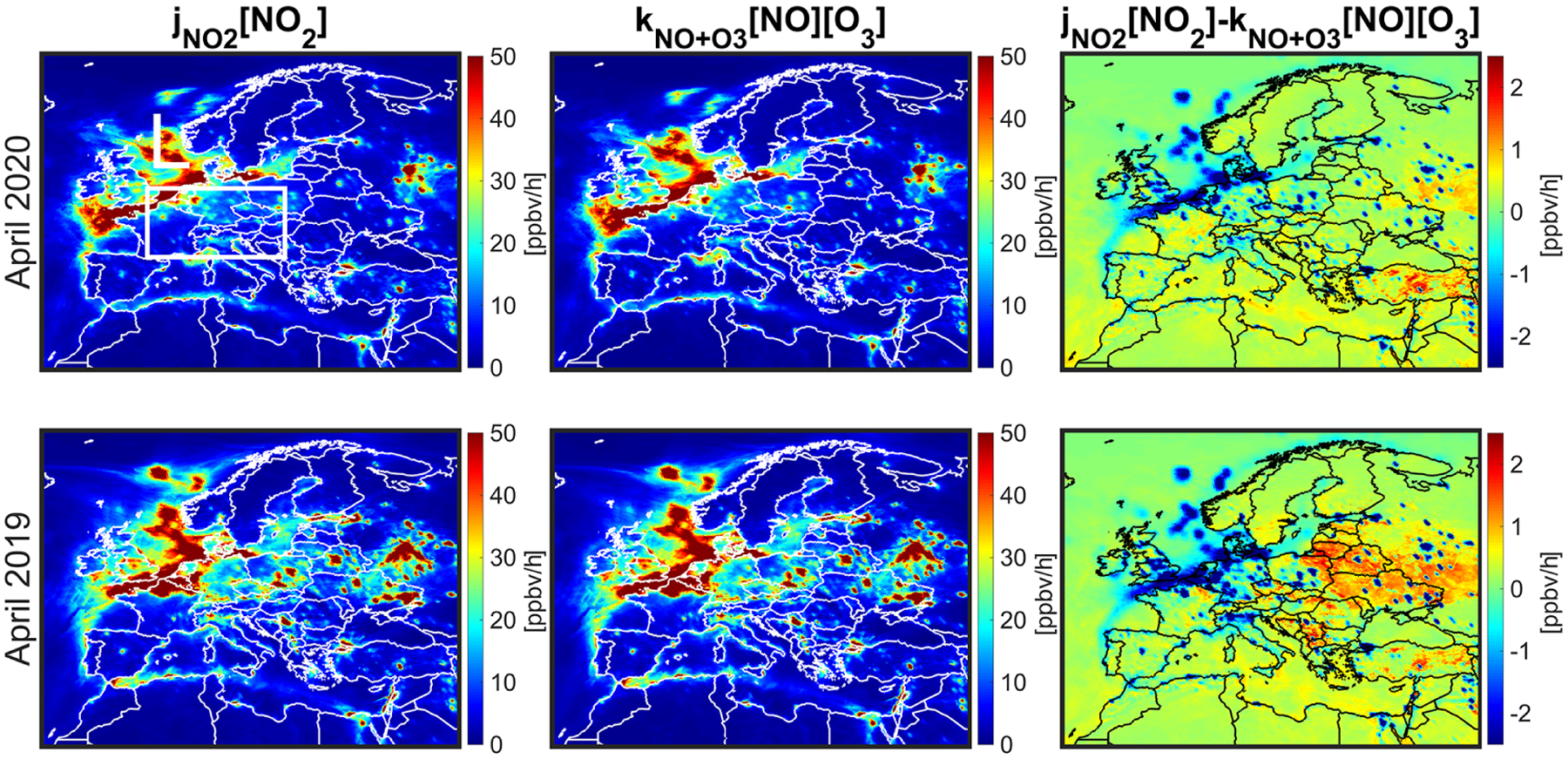
Surface chemical processes involved in Eq. (6) (ppbv h^−1^) pertaining to the O_3_–NO–NO_2_ partitioning in April 2020 and 2019 during MDA8 hours. The constrained model by the satellite observations is used to derive these outputs.

Figure 11 displays the reactions rates of each individual component involved in Eq. (6) averaged during the MDA8 hours. HO_2_+NO is the dominant chemical source of ozone correlating well with the changes in NO_*x*_ and prevailing chemical conditions regimes (NO_*x*_-sensitive vs. VOC-sensitive ones). Souri et al. (2020a) found the reaction of RO_2_+NO to be primarily dependent on VOCs. Likewise, we observe a strong degree of correlation between the anomaly of RO_2_+NO and that of VOCs (Figs. S1 and S2). Figure 11 indicates that the chemical pathways of ozone loss are rather constant between the two years; therefore, the largely negative PO_3_ over urban areas shown previously in Fig. 10 is not reproducible using this equation. Figure 12 shows the reactions rates of $J_{{\text{NO}}_{2}}\left({\text{NO}}_{2}\right)$, $k_{\text{NO}+{\text{O}}_{3}}(\text{NO})\left({\text{O}}_{3}\right)$, and the difference during the MDA8 hours. The difference maps replicate the largely negative PO_3_ over cities suggesting that we are not in the diel steady state, and O_3_ titration is prevailing due to relatively low photochemistry in the springtime. Table 2 lists the averaged reactions rates involved in Eqs. (6) and (7) along with the numerically solved PO_3_ shown in Fig. 10 over box L. These numbers suggest that the major chemical pathways of enhanced ozone are through $J_{{\text{NO}}_{2}}\left({\text{NO}}_{2}\right)$ and $k_{\text{NO}+{\text{O}}_{3}}(\text{NO})\left({\text{O}}_{3}\right)$, implying that O_3_–NO–NO_2_ partitioning is more consequential than other chemical pathways. This analysis strongly coincides with Lee et al. (2020) and Wyche et al. (2021) who observed roughly constant O_3_ + NO_2_ concentrations over the UK before and during the 2020 lockdown.

## 4 Summary

The slowdown in human activities due to the COVID-19 pandemic had a large impact on air pollution over Europe (Barré et al., 2021; Sicard et al., 2020; Sun et al., 2021). Satellite monitoring systems with large spatial coverage help shed light on the spatial and temporal extent of those impacts. The relationships between satellite-derived columns and near-surface emissions have proven difficult to fully establish without using realistic models, capable of providing insights on the convoluted processes involving chemistry, dynamics, transport, and photochemistry and therefore help with deciphering what anomaly maps of satellite concentrations are suggesting (e.g., Goldberg et al., 2020). To address these challenges, we jointly constrained NO_*x*_ and VOC emissions using TROPOMI HCHO and NO_2_ columns following a non-linear Gauss Newton method developed in Souri et al. (2020a), in addition to assimilating MODIS AOD observations based on J. Jung et al. (2019). The constrained emissions also permitted investigating the simultaneous effects of physiochemical processes contributing to ozone formation, illuminating the complexities associated with non-linear chemistry.

Several implications of the derived emissions for the months of March, April, and May 2020 (lockdown) relative to those in 2019 (baseline) were investigated. First, as previously reported (Sicard et al., 2020; Barré et al., 2021), we observed a significant reduction in NO_*x*_ in March (14 %–31 %) in several major polluted regions including Paris, London, Madrid, and Milan. The reductions were further seen in other cities such as Rome, Brussels, Frankfurt, Warsaw, Belgrade, Kyiv, and Moscow (34 %–51 %) in April. Second, NO_*x*_ emissions decreased drastically in April rather than March in the UK, Moscow, and Poland due to the timeline of restrictions. Third, the changes in NO_*x*_ suggested by TROPOMI NO_2_ and the constrained model over northeastern Germany in March and central and eastern Europe in May were unrealistic, possibly due to observations or the model issues. Fourth, we observed a weak observational constraint on VOC emissions from TROPOMI HCHO except for lower latitudes whose values were dictated by air temperature.

The constrained model calculations gave good representations of near-surface NO_2_ changes in April (model: − 21 ± 17 %, observation: −29 ± 21 %) in places where the top-down estimates are strongly constrained by TROPOMI (averaging kernels > 0.5), but inferior representations in other months, especially in May (model: −12 ± 18 %, observation: −25 ± 20 %). This tendency mainly arose from TROPOMI observation frequencies; too few days (10 %–26 % out of a month) in May due to cloudiness precluded the determination of realistic NO_*x*_ emission changes.

We observed surface MDA8 ozone increase from both model and measurements in April 2020 with respect to the baseline. Comparisons of calculation by the constrained model in terms of MDA8 surface ozone found a reasonable agreement with observations in the proximity of central Europe in April (model: +3.73 ± 3.94 %, +1.79 ppbv, observation: +7.35 ± 11.27 %, + 3.76 ppbv). These comparisons indicate that the performance of the constrained model to reproduce the ozone enhancement feature is promising, suggesting fruitful information in TROPOMI, although reasons behind the underestimation of the enhancement remained unexplained. It was clear that the dominantly negative ozone production rates dictated by O_3_–NO–NO_2_ partitioning $\left(J_{{\text{NO}}_{2}}\left[{\text{NO}}_{2}\right]-k_{\text{NO}+{\text{O}}_{3}}[\text{NO}]\left[{\text{O}}_{3}\right]\right)$ became less negative primarily due to the reduced NO_*x*_ emissions in urban areas where O_3_ titration occurred. This tendency was in agreement with studies of Lee et al. (2020) and Wyche et al. (2021). We found negligible differences in ozone production from [HO_2_+RO_2_][NO] and ozone loss from O^1^D+H_2_O and O_3_+HO_*x*_ between the two years suggesting photochemistry was rather low in the springtime over Europe.

We further quantified the contributions of physical processes (transport, diffusion, and dry deposition) and chemistry to the formation/loss of ozone using the integrated process rates. The physical processes decreased MDA8 ozone by −4.83 ppbv resulting from relatively larger dry deposition in 2020, whereas chemistry (ozone production) augmented ozone levels by +5.89 ppbv, indicating that rising ozone was primarily impacted by changes in chemistry. Enhanced air temperature and photolysis in 2020, both of which were well captured in our model, also affected chemistry. Experiments with fixed anthropogenic emissions underwent significant enhancement in surface MDA8 ozone over central Europe, but those only contribute to 42 % of the total enhancement indicating that anthropogenic emissions were the major factor.

The results shown here reveal previously unquantified characteristics of ozone and its precursors emission changes during the 2020 lockdown in Europe. We have been able to measure the amount of changes along with the level of confidence in NO_*x*_ (and partly VOC emissions) using a state-of-the-art inversion technique by leveraging satellite observations, which in turn, allowed us to unravel the physiochemical processes contributing to increased ozone in Europe. Unless a comprehensive air quality campaign targeting COVID-19-related lockdown is available, we recommend that the impact of lockdown on air pollution should be examined through the lens of well-established models constrained by publicly available data, especially those from space in less cloudy environments.

## Supplementary Material

Supplement1

## Figures and Tables

**Table 1.** Statistics reported in several validations studies comparing TROPOMI tropospheric NO_2_ against independent observations.

Study	Location	Time period	Benchmark instrument	Bias (low)	Dispersion	Modification	Modified bias (low)
Chan et al. (2020)	Munich	May 2018–Apr 2019	MAX-DOAS	30 %	NA	In situ MAX-DOAS profiles	17 %
Griffin et al. (2019)	Canadian oil sands	Mar–May 2018 (vl.01)	Pandora (direct Sun)	15 %–30 %	NA	Higher-resolution profiles (10 km) and albedo	0 %–25 %
Judd et al. (2020)	New York	Jun–Sep 2018	GeoTASO	19 %–33 %	NA	Higher-resolution profiles (12 km)	7 %–19 %
Verhoelst et al. (2021)	Global	Apr 2018–Feb 2020	MAX-DOAS	37 % (average), 23 %–51 % (range)	3.5 × 10^15^ molec/cm^2^	NA	NA
P. Wang et al. (2020)	Atlantic and Pacific oceans	Four campaigns during Dec 2018–Jul 2019	MAX-DOAS	Negligible	NA	NA	NA
Zhao et al. (2020)	Greater Toronto area	Mar 2018–Mar 2019	Pandora (direct Sun)	24 %–28 % (suburban/urban) +4 %–10 % (rural)		Higher-resolution profiles (10 km) and albedo	13 %–24 % (suburban/urban) +14 %–15 % (rural)

NA – not available

**Table 2.** Reaction rates relating to the chemical pathways of ozone formation and loss over box L (proximity of central Europe).

Reactions	Production or loss (L)	April 2020 (ppbv/h)	April 2019 (ppbv/h)	Net diff* (ppbv/h)
HO_2_ + NO	P	0.85	0.91	−0.06
RO_2_ + NO	P	0.44	0.41	+0.03
NO_2_ + OH	L	0.10	0.14	+0.04
O^1^D + H_2_O	L	0.07	0.08	+0.01
O_3_ + VOCs	L	0.01	0.01	0.00
O_3_ + HO_*x*_	L	0.09	0.08	−0.01
$J_{{\text{NO}}_{2}}\left[{\text{NO}}_{2}\right]$	P	14.61	27.28	−12.67
$k_{\text{NO}+{\text{O}}_{3}}[\text{NO}]\left[{\text{O}}_{3}\right]$	L	15.11	28.52	+13.40
$J_{{\text{NO}}_{2}}\left[{\text{NO}}_{2}\right]-k_{\text{NO}+{\text{O}}_{3}}[\text{NO}]\left[{\text{O}}_{3}\right]$	n/a	−0.50	−1.24	+0.74
Numerically solved PO_3_	n/a	−0.79	−1.53	+0.74

* A positive net difference indicates higher (lower) production (loss) in 2020 with respect to 2019.

n/a – not applicable

## Data Availability

The atmospheric inversion data are publicly available from Souri et al. (2021a, https://doi.org/10.17632/jchfxsrvsb.1). The model outputs are available upon the request from ahsouri@cfa.harvard.edu. The links on where to download surface and satellite observations that are used in this study are already provided in the text.
