# Supplementary material for: Unraveling pathways of elevated ozone induced by the 2020 lockdown in Europe by an observationally constrained regional model using TROPOMI
Source: Atmos Chem Phys. Author manuscript; Available in PMC 2022 Dec 16. (PMC8721815; doi:10.5194/acp-21-18227-2021)
Supplement: Supplement1 [file NIHMS1767504-supplement-Supplement1.pdf]

Supplement of Atmos. Chem. Phys., 21, 18227–18245, 2021
https://doi.org/10.5194/acp-21-18227-2021-supplement
© Author(s) 2021. CC BY 4.0 License.

Atmospheric Chemistry and Physics
Open Access

Supplement of

Unraveling pathways of elevated ozone induced by the 2020 lockdown in Europe by an observationally constrained regional model using TROPOMI

Amir H. Souri et al.

Correspondence to: Amir H. Souri (ahsouri@cfa.harvard.edu)

The copyright of individual parts of the supplement might differ from the article licence.

Table of Contents

S1. TROPOMI HCHO 3
Figure S1. 5
S2. Anomaly of HCHO columns and top-down VOC emissions 6
Figure S2. 7
Figure S3. 8
Figure S4. 9
Figure S5. 10
Figure S6. 11
Figure S7. 12
Figure S8. 13
Figure S9. 14
Figure S10. 14
Figure S11. 15
Figure S12. 15
Figure S13. 16
Figure S14. 17
Figure S15. 18
Figure S16. 19
Figure S17. 20
Figure S18. 21
Figure S19. 21
Table S1. 22
Table S2. 22
Table S3. 23
Table S4. 23
References 24

S1. TROPOMI HCHO

We use daily offline S5P TROPOMI HCHO total slant columns [Copernicus Sentinel data processed by ESA, German Aerospace Center (DLR), 2019]. A full description of the algorithm can be found in De Smedt et al. [2018]. The HCHO products for the study time period were produced by processor versions v01.01.05 (1 March 2019 – 28 March 2019), v01.01.06 (28 March 2019 – 23 April 2019) and v01.01.07 (23 April 2019 onward). The newer versions have added updates to the surface classification climatology and cloud products that might have some effects on the magnitude of HCHO in cloudy scenes. We again remove bad pixels based on qa_flag < 0.75 and recalculate shape factors using the simulated profiles derived from our regional model.

Validation efforts reported in the sixth Quarterly Validation Report of the Copernicus Sentinel-5 Precursor Operational Data Products [Lambert et al., 2020] indicate varying biases depending on the magnitude of HCHO concentrations in comparison to ground-based observations. Locations with HCHO concentrations above 8×10^{15} molec/cm^2 show a low bias of ~-31%. Conversely, clean sites with HCHO concentrations below 2.5×10^{15} molec/cm^2 undergo a high bias of 26%. Vigouroux et al. [2020] expanded the validation suite by including more than 25 FTIR stations located over both pristine and polluted sites. Results from the comparison with FTIR measurements (over clean areas) also indicate a high bias, whereas those compared in polluted areas show a low bias. By compiling numbers quoted in Lambert et al. [2020] and Vigouroux et al. [2020], we correct the existing biases in TROPOMI HCHO by scaling 25% (<2.5×10^{15} molec/cm^2) down columns in clean areas and 30% (>=8×10^{15} molec/cm^2) up in polluted areas. We assume the constant term of errors (e_{const}) to be equal to 4% of HCHO total columns based on Vigouroux et al. [2020]. The precision error ($e_{precision}$) is populated with the column uncertainty variable provided with the data.

We investigate the changes in HCHO total columns shown in Figure S1. Various VOCs with different sources contribute to the formation of HCHO (see Figure 2 in Chan Miller et al. [2016]). In theory, it is easier to single out anthropogenic-derived HCHO concentration by HCHO measurements made in wintertime, although temperature and photochemistry are always key influencers of oxidizing/photolyzing all types of VOCs. The inevitable trade-off for this is dealing with a weaker signal that is near to instrument detection limit. The TROPOMI HCHO retrieval offers a low detection limit for individual pixels (7×10^{15} molec/cm^2) that can be further lowered down by co-adding measurements (roughly a factor of $1/\sqrt{n}$). Accordingly, we observe a promising

signal in March over eastern European countries that is not explainable by biogenic emissions; but the magnitudes of the difference over these areas (<1.5×10^{15} molec/cm^2) are below the detection limit (~ 2.4×10^{15}molec/cm^2 given the co-added measurements over time).

In April, results show elevated HCHO concentrations in high latitudes in 2019 (box I), mainly a result of biomass burning activities in eastern Europe [e.g., Karlsson et al. 2013; https://earthobservatory.nasa.gov/global-maps/MOD14A1_M_FIRE, accessed June 2020]. As temperature rises in May, the footprint of biogenic emissions become more visible. This signal is not only induced by the inherent temperature-dependency of biogenic emissions, but also stems from faster isoprene oxidation through higher levels of OH [Pusede et al. 2015]. The dipole anomaly of HCHO columns suggested by TROPOMI (box J and K) pertains largely to variations in ambient surface air temperature (discussed later).

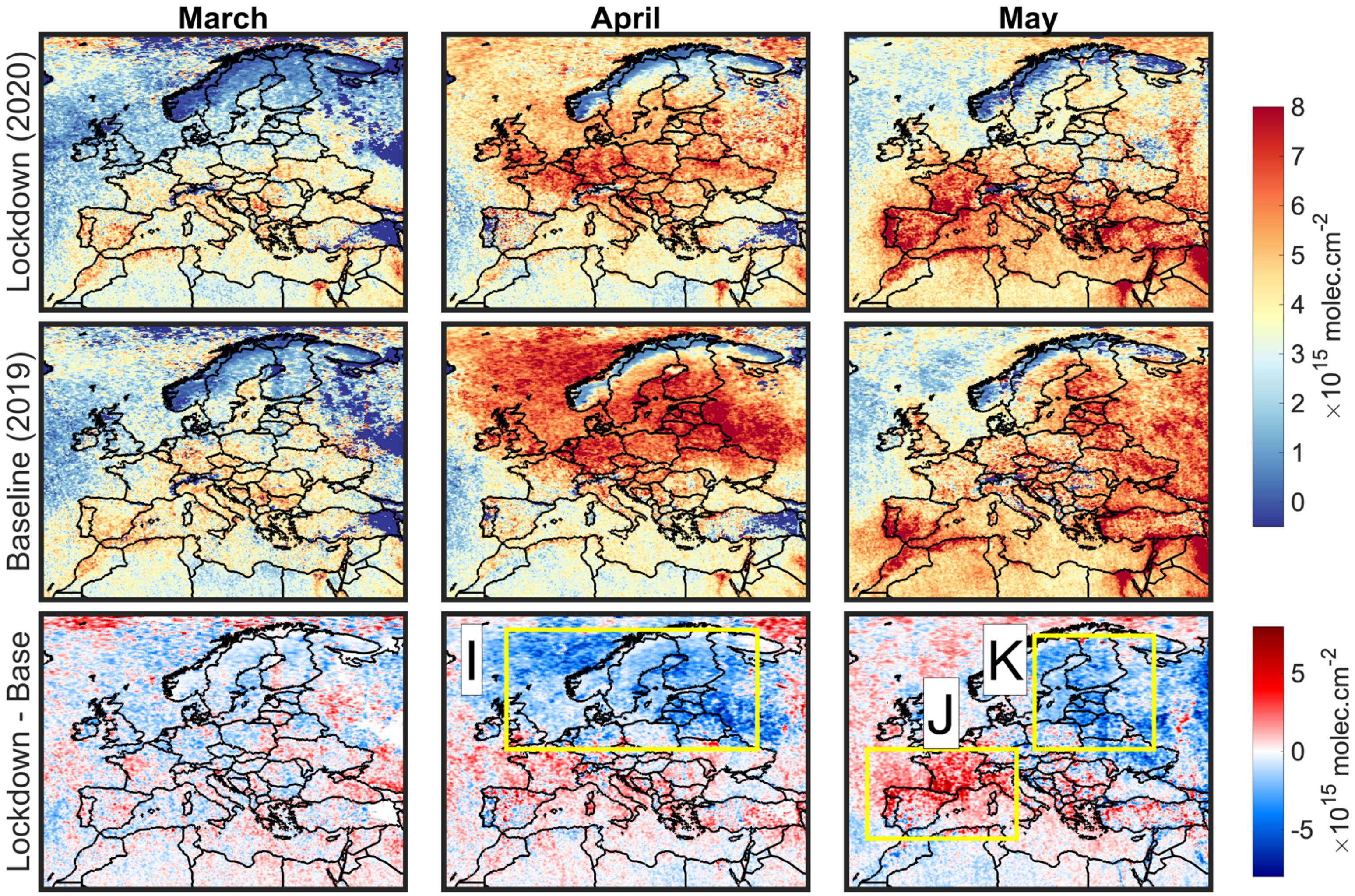

Figure S1. (first row) Maps of HCHO column from the TROPOMI sensor during months of March, April, and May in 2020 (lockdown). (second row) Same as the first row but for the baseline year (2019). (last row), Difference of the columns in 2020 with respect to those of 2019. All columns are corrected for the bias and their AMFs are recalculated iteratively based on the posterior profiles derived from our inverse modeling practice. The satellite-derived columns are subject to errors, so a direct interpretation of their magnitudes cannot be performed in a robust manner.

S2. Anomaly of HCHO columns and top-down VOC emissions

As to VOC emissions, we observe improvements in the magnitude and spatial distribution of simulated HCHO columns after the inversion with respect to TROPOMI data over areas with a practical amount of information (e.g., AK>0.2) (Figure S15 and S16). Very low averaging kernels over major European cities in this month are indicative of inadequacies of one-month averaged TROPOMI HCHO data in March. The inversion partly corrects for the large underrepresentation of biomass burning emissions in high latitudes occurring in April 2019 but due to large uncertainties of the retrieval over this area, averaging kernels are low. Vigouroux et al. [2020] showed FTIR HCHO columns to be around 4-6×10^{15} molec/cm^2 in Saint Peterburgh (59.9^{o}N), Kiruna (67.8^{o}N), and Sodankylä (67.4^{o}N) in April 2019. Despite some improvements over the biomass burning areas in April 2019, the model still greatly underestimate HCHO columns suggesting more precise observations are needed to adjust the emissions. It is worth noting that the TROPOMI bias-correction factors used here based on Vigouroux et al. [2020] are not necessarily correct over this area possibly due to snow cover, the profile shapes, or non-linear aerosol impacts on AMFs (see Figure5 in Vigouroux et al. [2020]). The predominately high pressure system formed over these areas in April 2019 (Figure S17) impedes the transport of the biomass burning pollution to central Europe. The inversion suggests larger VOC emission rates in April 2020 compared to April 2019 over central Europe. Ordóñez et al. [2020] reported ambient temperature along with solar radiation to be higher than the norm. This is primarily due to a well-developed high-pressure system over the region (Figure S17) resulting in elevated HCHO columns. The top-down estimate is indicative of too low prior VOC emission rates over this area in April 2020. However, the reason behind the enhancement of VOCs over several urban areas such Paris and Po Valley is not fully understood. This can be caused by the errors in the chemical mechanism or the limited VOC compounds provided by the CEDS emission inventory. Given the significant role of VOCs in the formation of ozone in urban settings, this correction with reasonable AK (~0.4) is crucial for precisely modeling the surface ozone anomalies (shown in the manuscript). We revisit the pronounced dipole anomaly of dominantly biogenic VOC emissions in May. In this month, the biogenic VOCs dominate. Our model suggests that ambient surface temperature differences between Russian and central Europe are more than 7°C, possibly inducing a strong dipole anomaly

in biogenic emissions. It is readily evident from the averaging kernels that more realistic information from TROPOMI HCHO is attainable in warmer months, contrary to the NO_2 case.

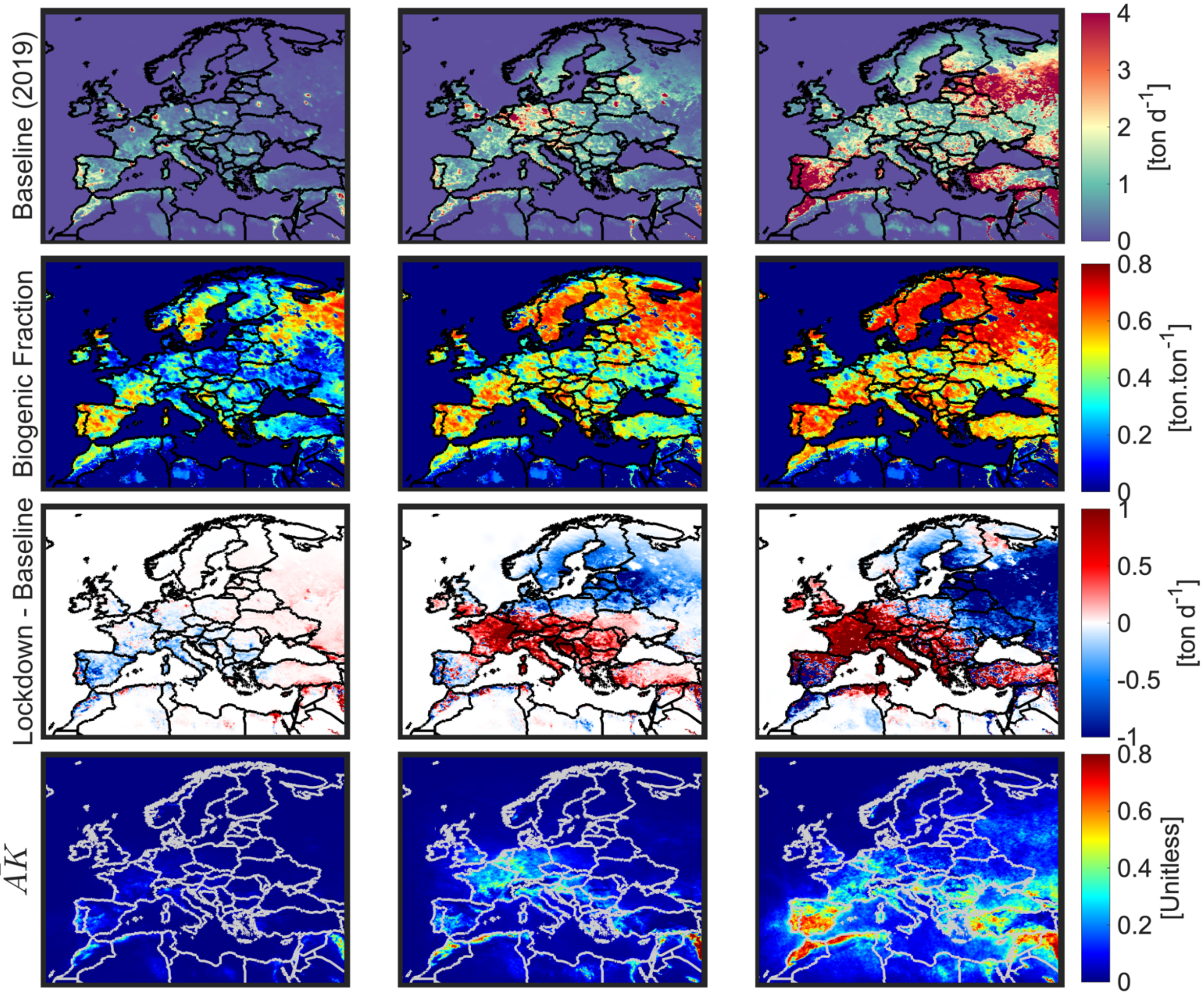

Figure S2. Top-down estimates of total VOC during months of March, April and May in 2019 (baseline) and the differences between emission in 2020 (lockdown) and 2019. To infer the magnitude of emissions in 2020, the second row should be added to the first one. Both TROPOMI HCHO and NO_2 observations are jointly used to estimate these numbers. Averaging kernels (mean values based on both 2019 and 2020 estimates) describe the level of credibility of the estimate which is heavily dependent on the TROPOMI signal-to-noise ratios. Biogenic fractions are based on the average values in 2019 and 2020.

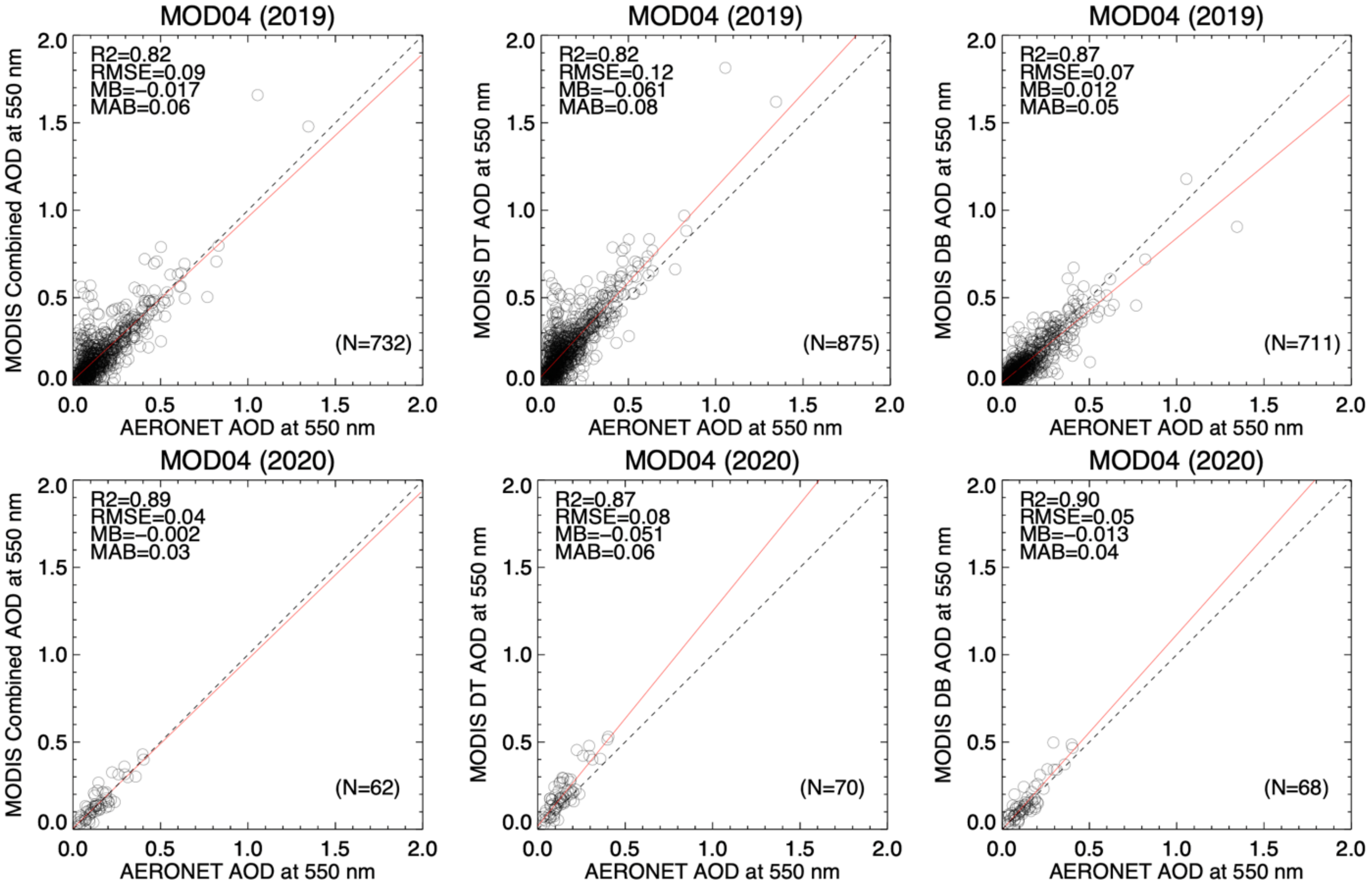

Figure S3. The comparison of MODIS AOD (on TERRA) and AERONET AOD observations over Europe for three different algorithms (combined, dark target and deep blue) in March-May 2020 and 2019.

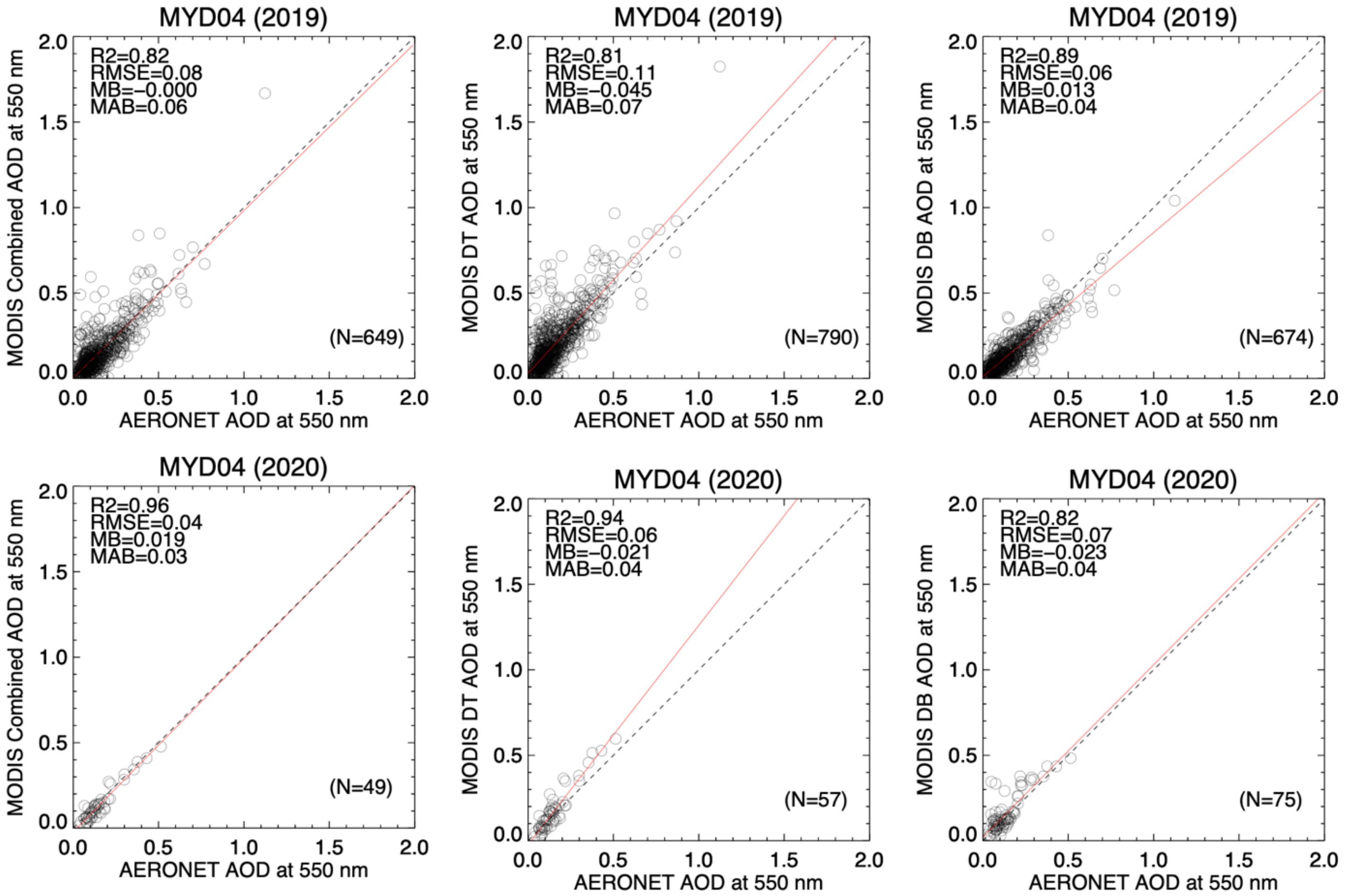

Figure S4. The comparison of MODIS AOD (on AQUA) and AERONET AOD observations over Europe for three different algorithms (combined, dark target and deep blue) in in March-May 2020 and 2019.

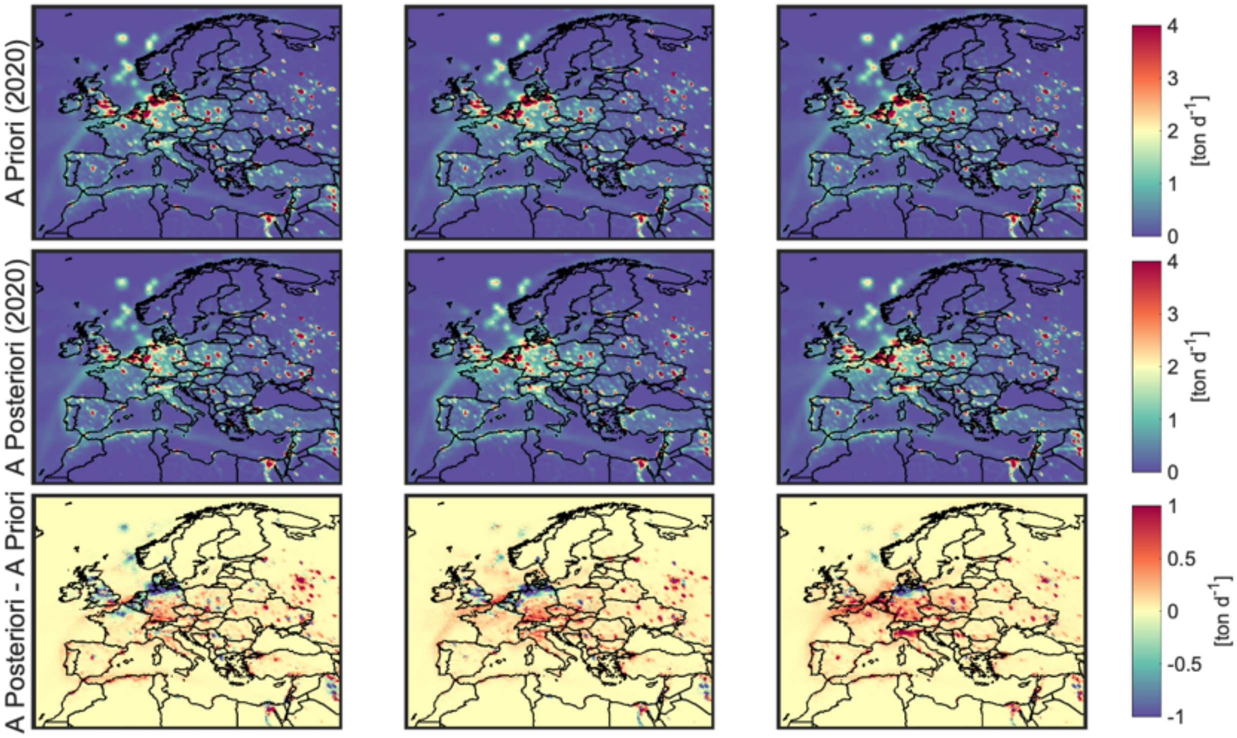

Figure S5. The a priori and the a postteriori of the total NOx emissions for the months of March (first column), April (second column), and May (last column) in 2020.

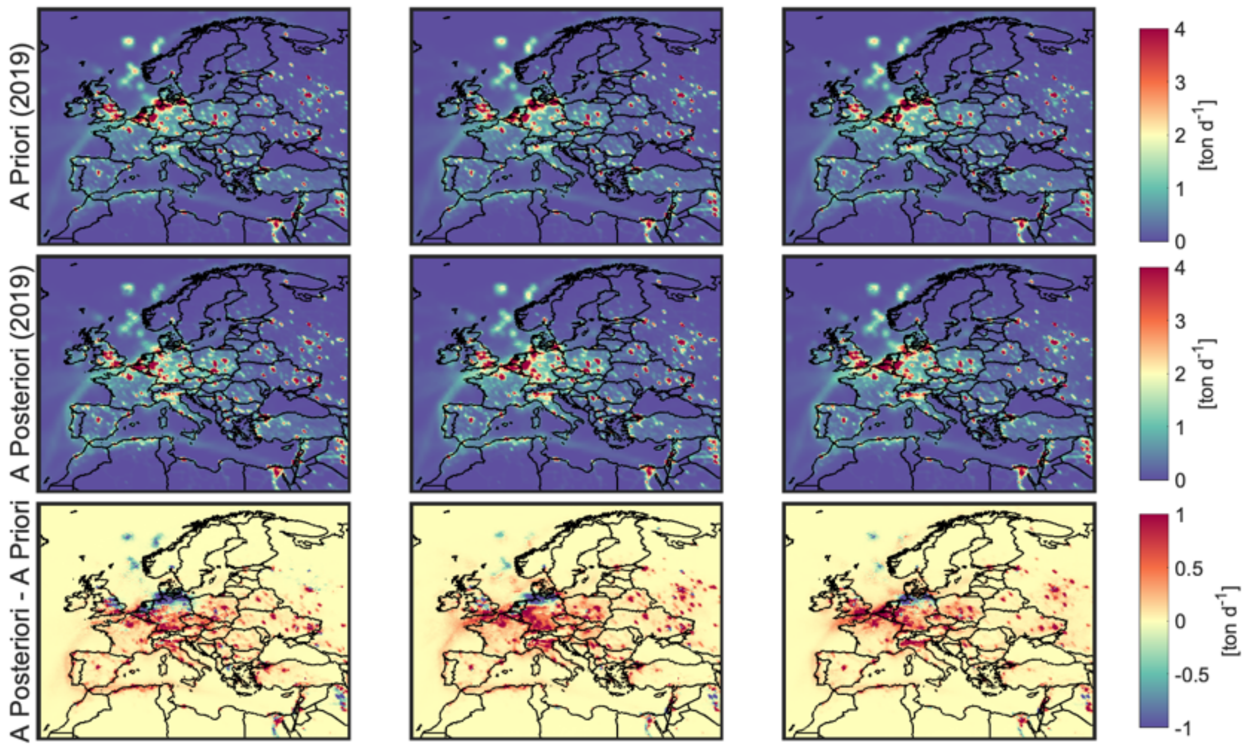

Figure S6. The a priori and the a postteriori of the total NO_x emissions for the months of March (first column), April (second column), and May (last column) in 2019.

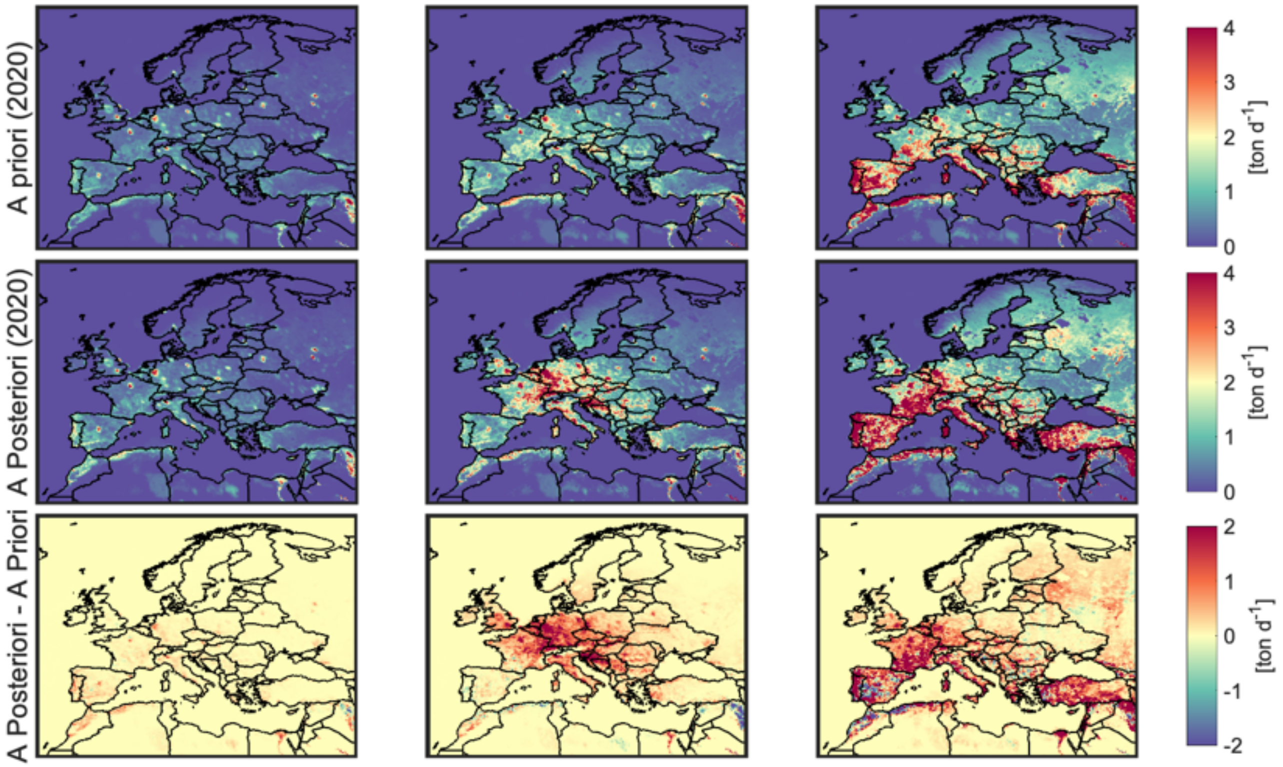

Figure S7. The a priori and the a posteriori of the total VOC emissions for the months of March (first column), April (second column), and May (last column) in 2020.

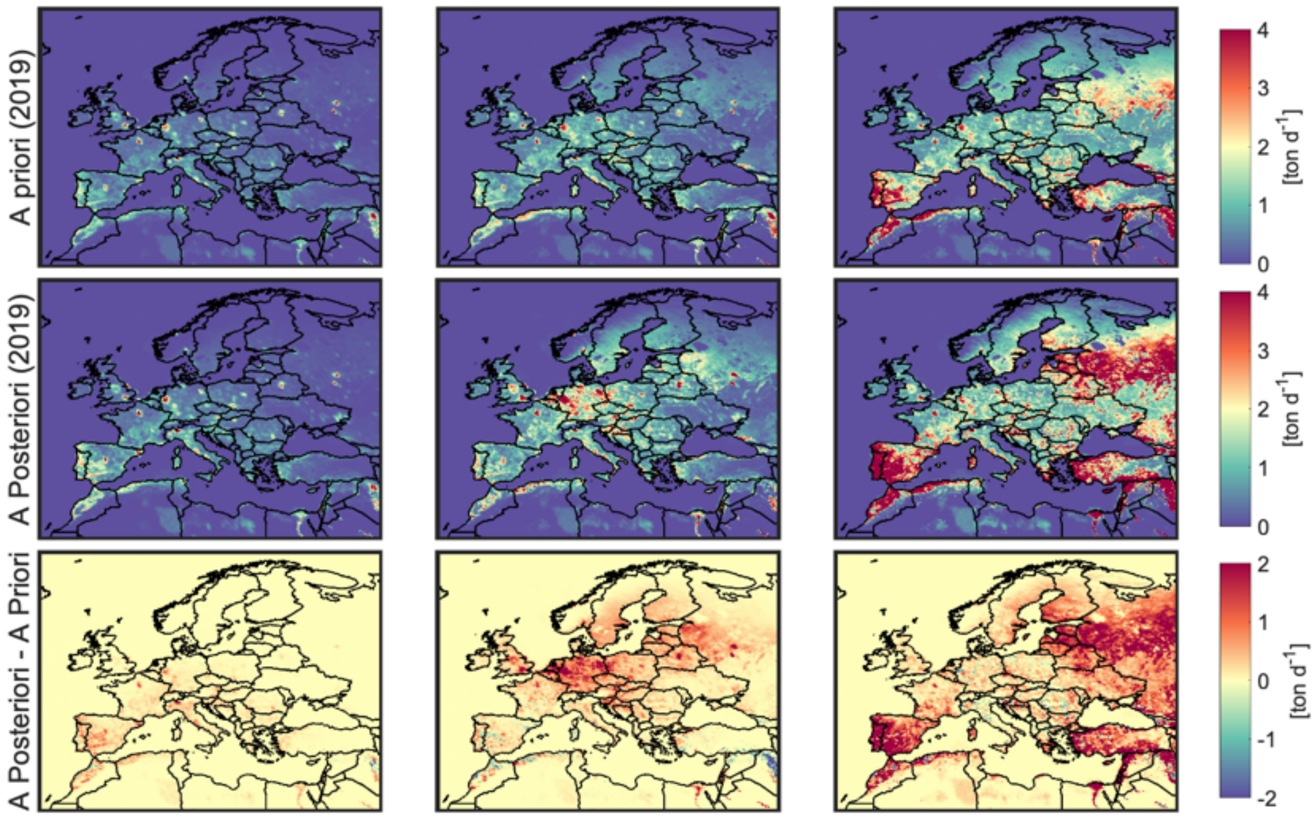

Figure S8. The a priori and the a postteriori of the total VOC emissions for the months of March (first column), April (second column), and May (last column) in 2019.

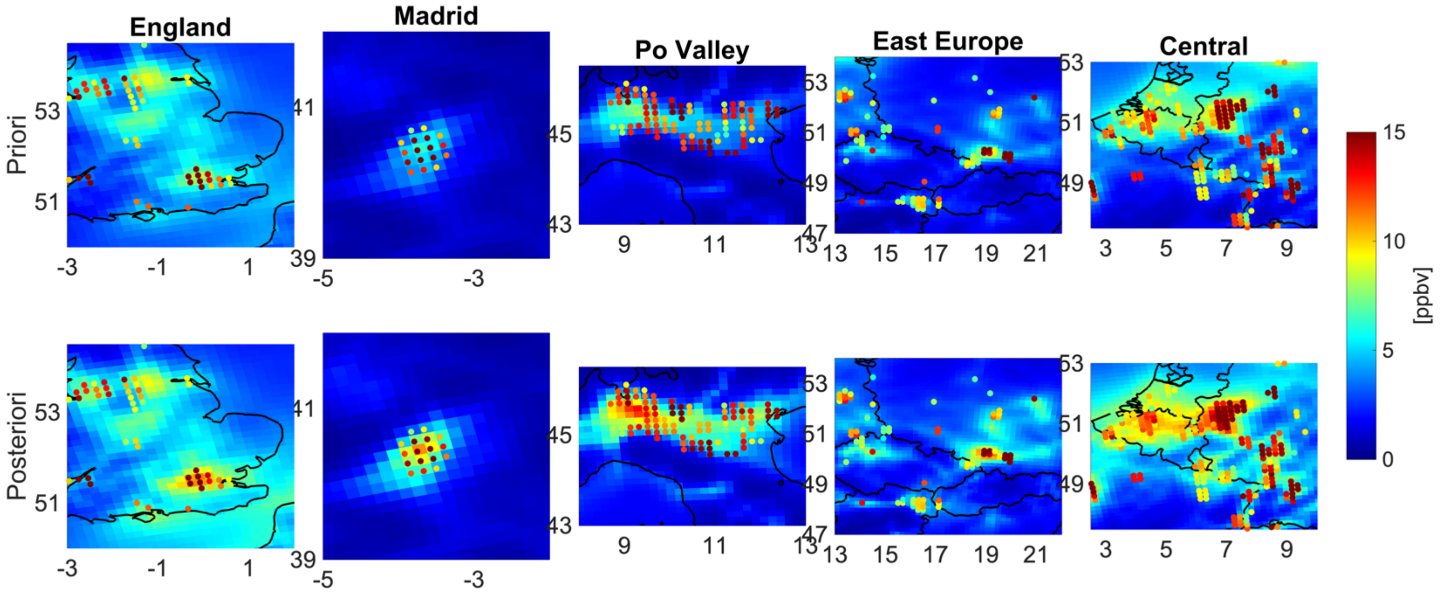

Figure S9. Comparison of daily-averaged surface NO_2 observations (circles) against the simulated model (map) in different regions around Europe in March-May 2019 (baseline). The first row uses the prior emissions whereas the second is based on the top-down emissions constrained by the satellite observations through an analytical non-linear inversion. All available observations within 15 km radius are averaged in each model grid cell.

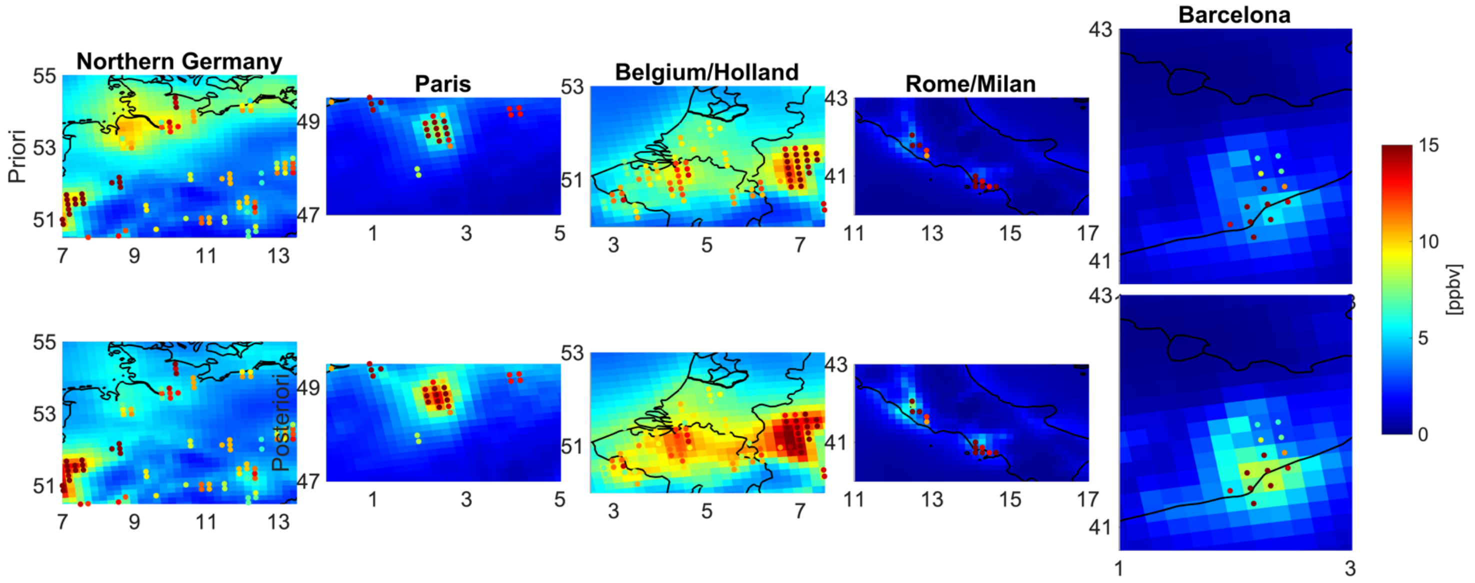

Figure S10. Similar to Figure S9 but in different areas.

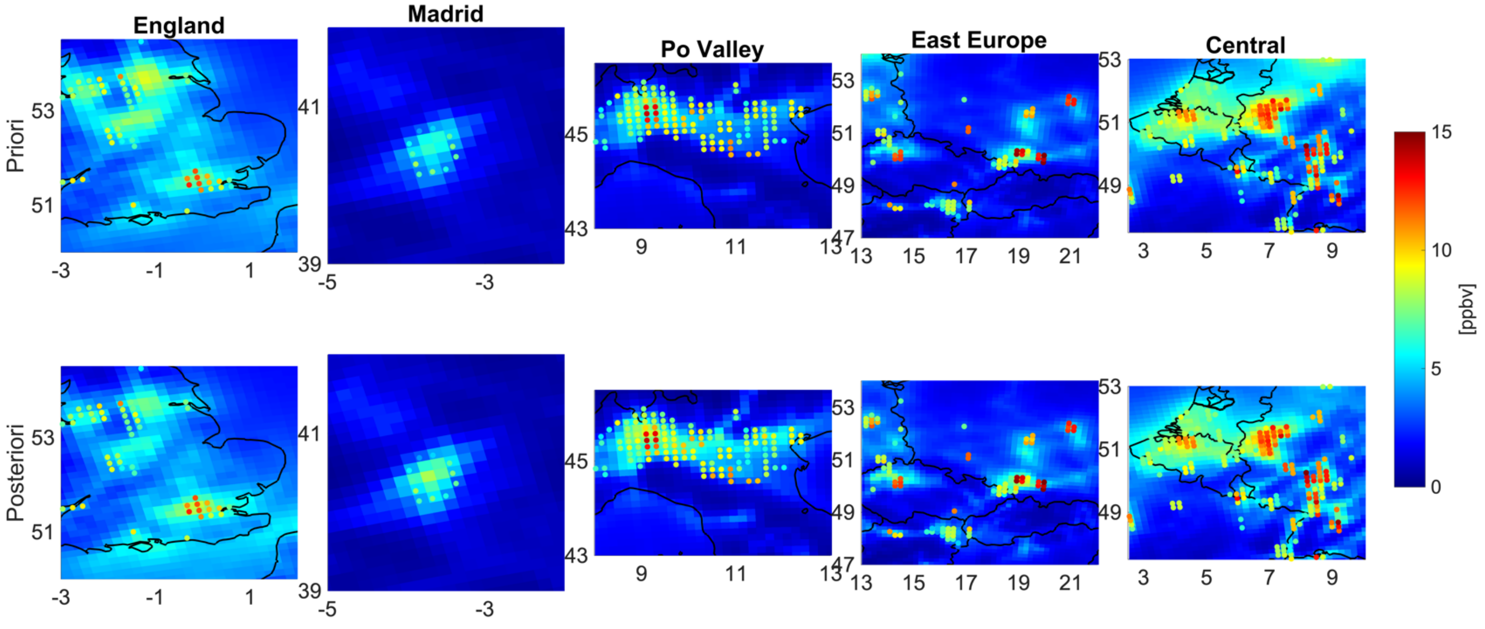

Figure S11. Comparison of daily-averaged surface NO_2 observations (circles) against the simulated model (contour) in different regions around Europe in March-May 2020 (baseline). The first row uses the prior emissions whereas the second is based on the top-down emissions constrained by the satellite observations through an analytical non-linear inversion.

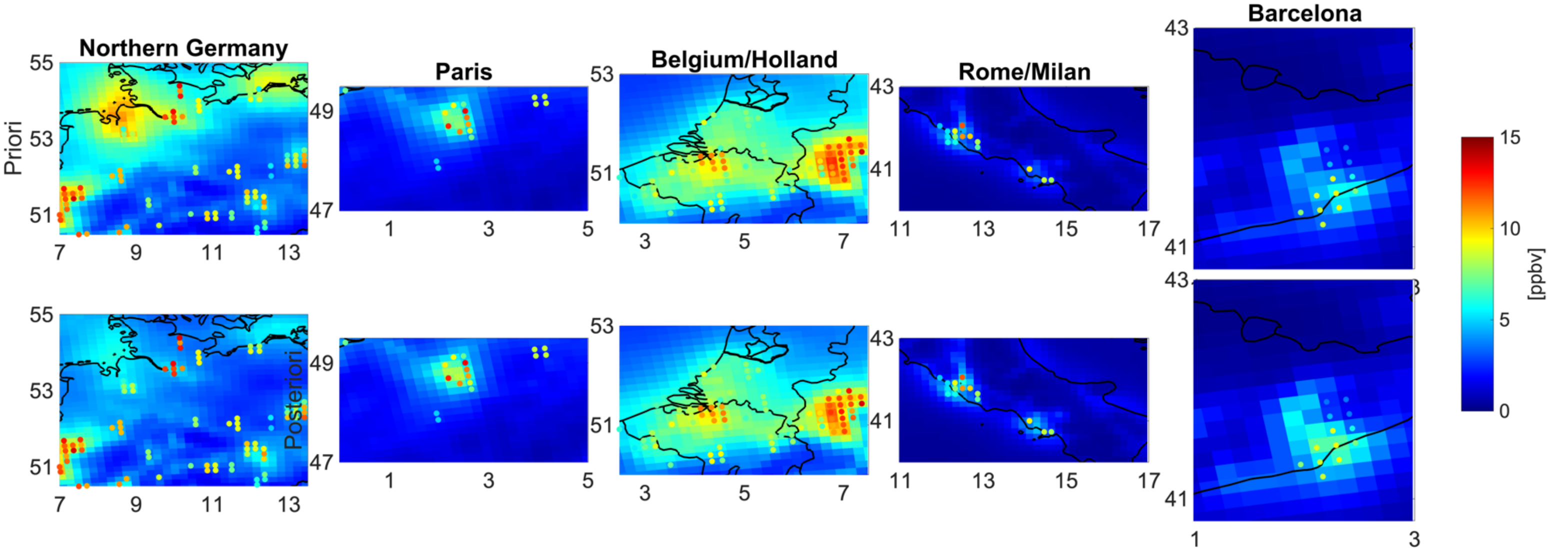

Figure S12. Similar to Figure S11 but for different areas.

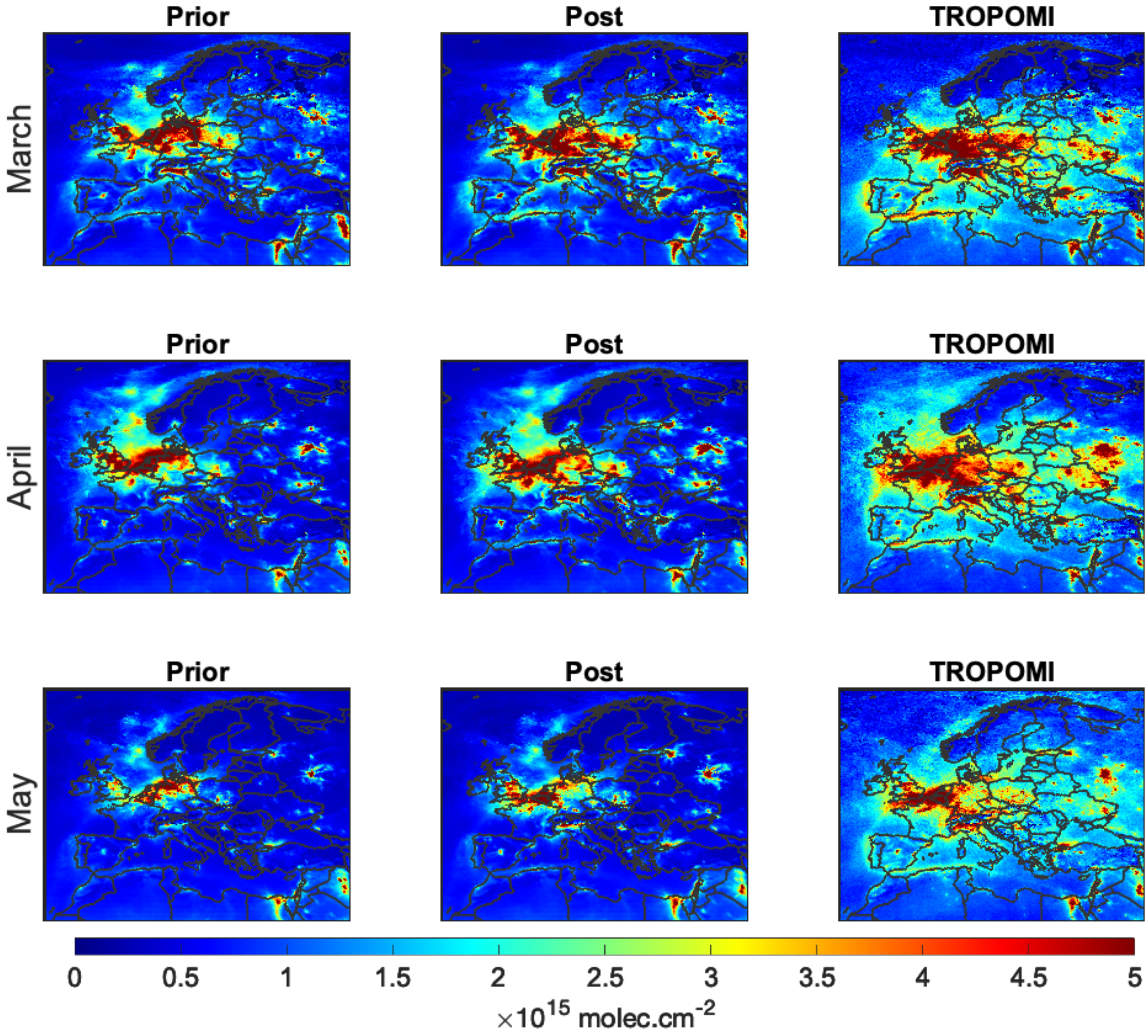

Figure S13. Comparison of simulated tropospheric NO_2 columns using the prior emission (prior) and top-down constrained emissions (post) with TROPOMI observations in **2019**. Note that TROPOMI columns are corrected for the bias and shape factors.

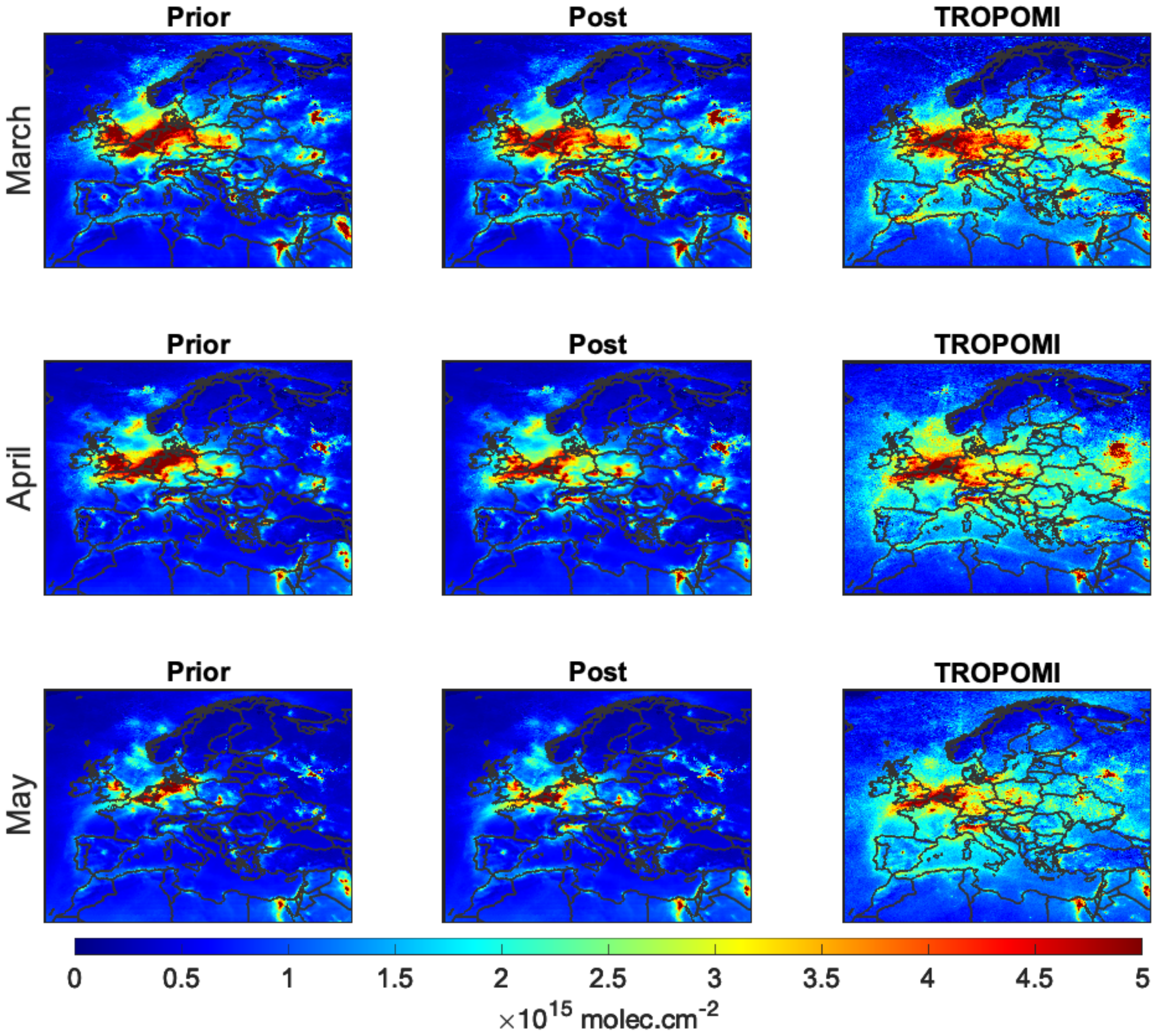

Figure S14. Same as Figure S13 but for 2020.

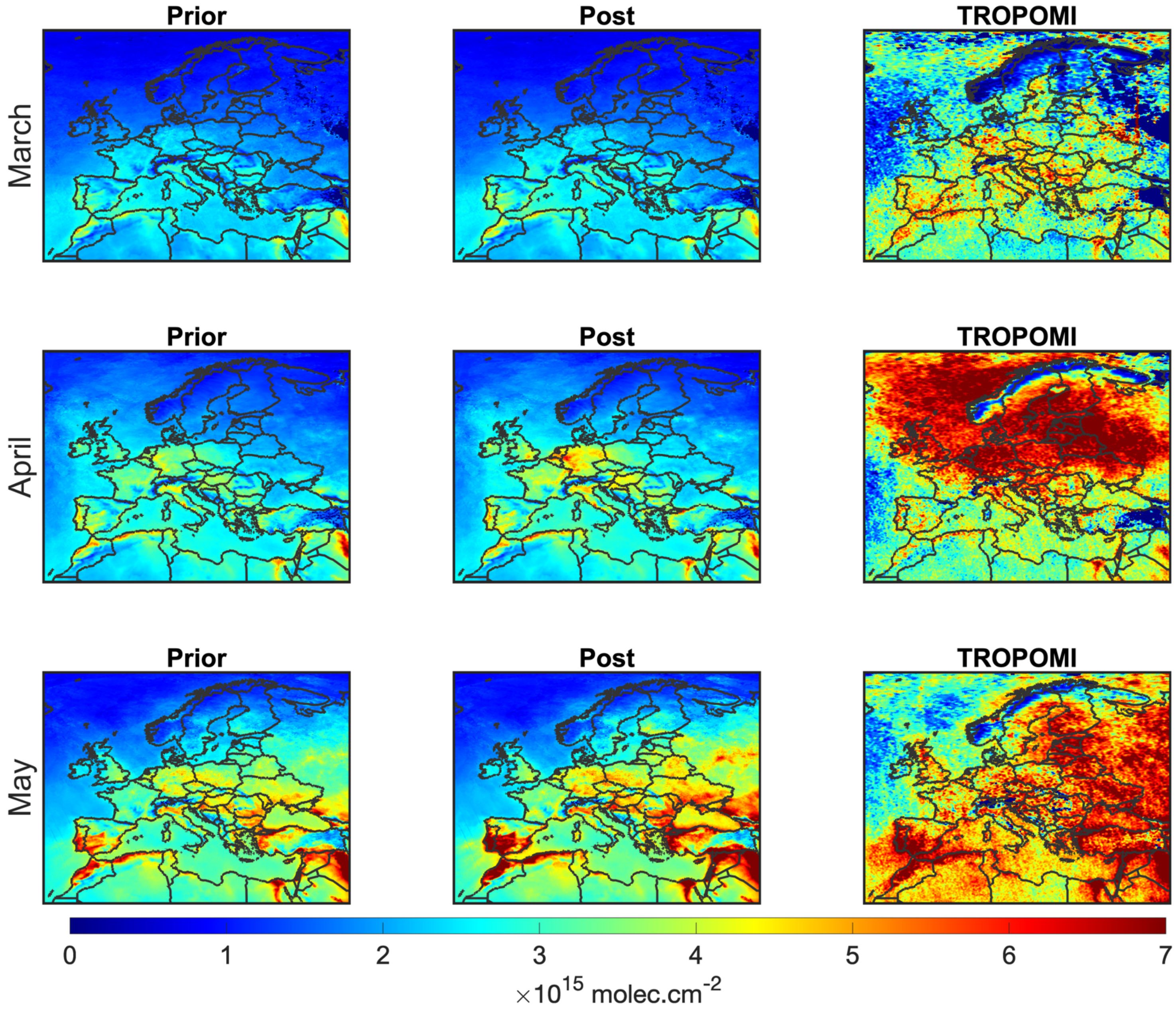

Figure S15. Comparison of simulated total HCHO columns using the prior emission (prior) and top-down constrained emissions (post) with TROPOMI observations in **2019**. Note that TROPOMI columns are corrected for the bias and shape factors.

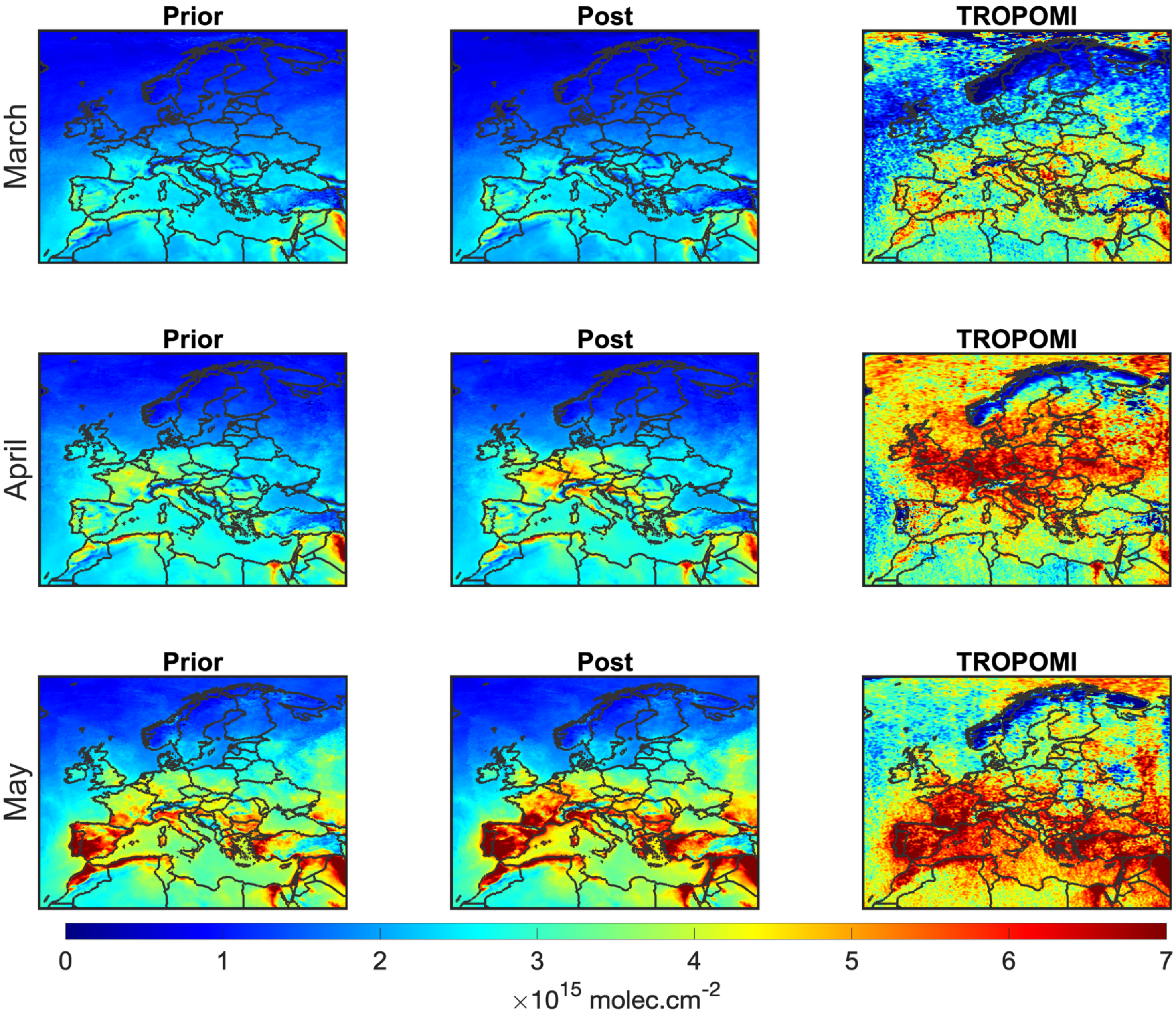

Figure S16. Same as Figure S15 but for 2020.

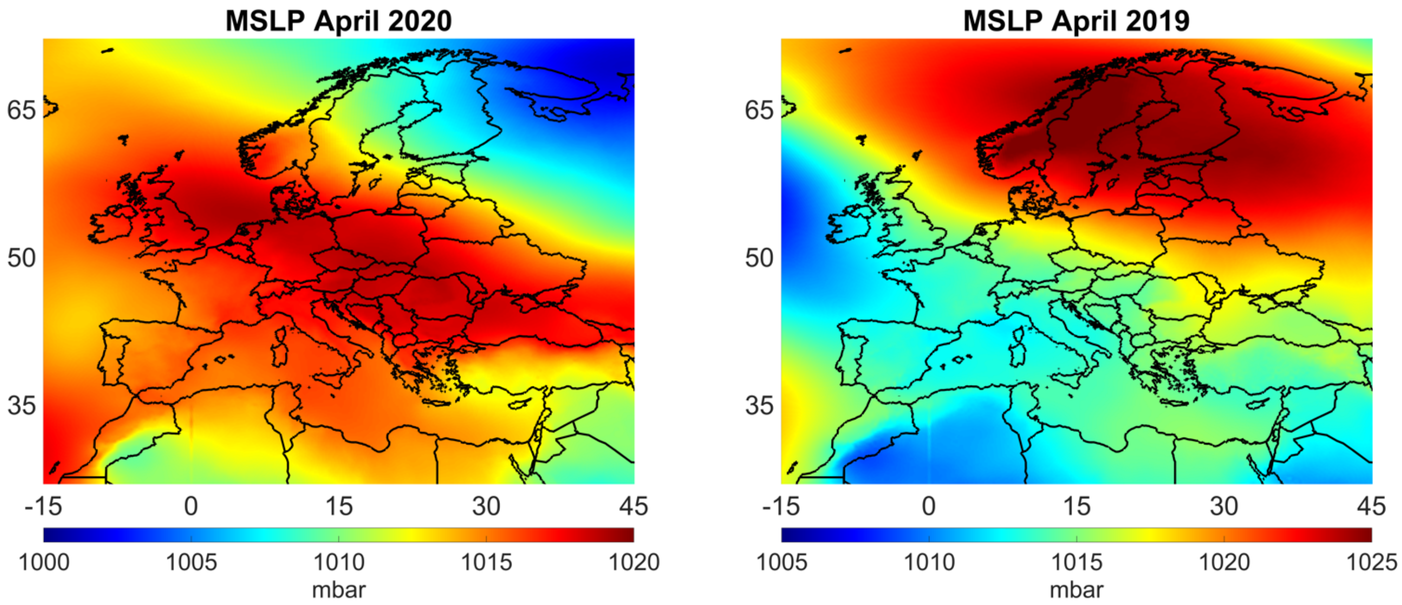

Figure S17. The WRF-simulated mean sea level pressures in April 2020 (left) and 2019 (right).

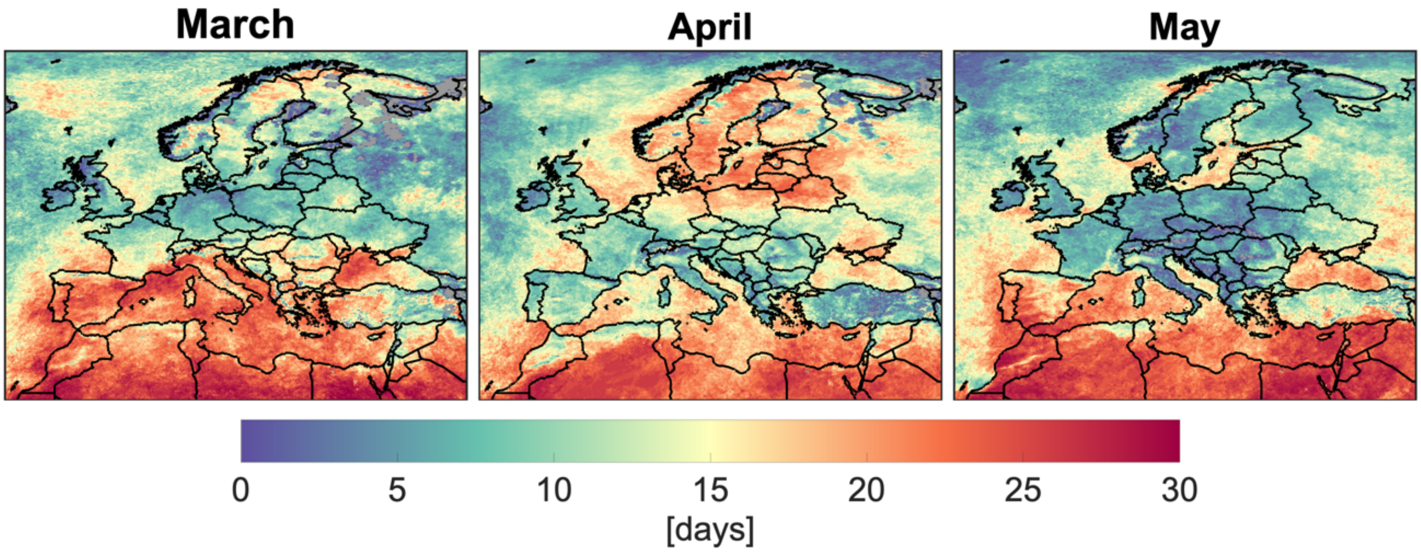

Figure S18. The number of good quality (qa_flag>0.75) TROPOMI tropospheric NO_2 days observed at 15×15 km^2 in 2019. These numbers are heavily affected by cloudiness.

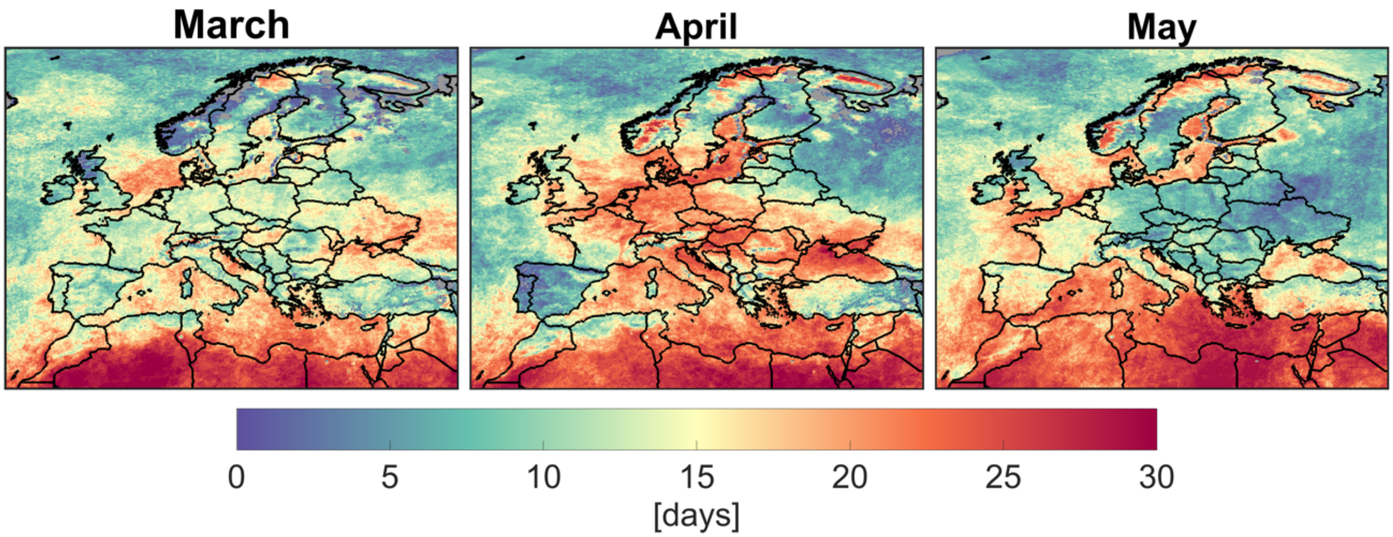

Figure S19. The number of good quality (qa_flag>0.75) TROPOMI tropospheric NO_2 days observed at 15×15 km^2 in 2020. These numbers are heavily affected by cloudiness.

Table S1. Statistics of surface temperature, relative humidity, and U/V-component wind from March through May in 2020.

Variables	Unit[a]	Corr[b]	$\mu_m \pm \sigma_m$[c]	$\mu_o \pm \sigma_o$[d]	MB[e]	MAE[f]	RMSE[g]
Temperature	℃	0.932	8.655±8.514	9.531±7.977	-0.876	2.281	3.200
Relative Humidity	%	0.687	70.06±23.61	66.930±21.665	3.125	12.151	18.266
U wind	ms^{-1}	0.806	0.541±3.331	0.549±3.580	-0.008	1.561	2.165
V wind	ms^{-1}	0.798	-0.153±3.285	-0.139±3.515	-0.014	1.573	2.172

Table S2. Statistics of surface temperature, relative humidity, and U/V-component wind from March through May in 2019.

Variables	Unit[a]	Corr[b]	$\mu_m \pm \sigma_m$[c]	$\mu_o \pm \sigma_o$[d]	MB[e]	MAE[f]	RMSE[g]
Temperature	℃	0.934	8.177±8.317	9.392±7.830	-1.216	2.356	3.214
Relative Humidity	%	0.728	75.18±21.82	69.02±21.368	6.159	12.86	17.075
U wind	ms^{-1}	0.806	0.448±3.059	0.498±3.642	-0.050	1.551	2.162
V wind	ms^{-1}	0.781	-0.078±2.928	-0.077±3.415	-0.001	1.550	2.150

[a]Units except for Correlation.

[c]Mean and 1σ standard deviation of Model variables (Y_m)

[d]Mean and 1σ standard deviation of observed variables (Y_o)

[e]Mean Bias ($\mu_m - \mu_o$)`

[f]Mean Absolute Error. $MAE = \frac{1}{n}\sum |Y_m - Y_o|$

[g]Root Mean Square Error. $RMSE = \frac{1}{n}\sqrt{\sum(Y_m - Y_o)^2}$

- Number of samplings : 5575860 for Temp, 5497687 for RH, 5073788 for wind.

Table S3. Statistics of the simulated surface NO_2 against the surface measurements in March-May 2019. The unit for MB, MAB, and RMSE is ppbv. MB is based on the model values minus the observed ones.

	'England'	'Madrid'	'Po Valley'	'East Europe'	'Central'	'Northern Germany'	'Paris'	'Belgium/Holland'	'Rome/Milan'	'Barcelona'
Corr (prior)	0.23	0.81	0.42	0.41	0.20	0.36	0.70	0.65	0.39	0.86
Corr (post)	0.42	0.82	0.48	0.43	0.26	0.52	0.72	0.70	0.41	0.92
MB (prior)	-6.36	-7.90	-7.63	-5.85	-5.93	-5.91	-9.41	-3.51	-10.20	-8.61
MB (post)	-5.14	-5.50	-5.33	-4.49	-3.58	-5.98	-6.56	-1.09	-8.91	-6.49
MAB (prior)	6.36	7.90	7.63	5.86	5.94	6.20	9.41	3.53	10.20	8.61
MAB (post)	5.14	5.50	5.35	4.71	3.98	5.98	6.56	1.86	8.91	6.49
RMSE (prior)	7.01	8.11	8.23	6.62	6.94	6.78	9.73	4.17	10.82	9.09
RMSE (post)	5.84	5.71	6.35	5.50	5.12	6.65	7.02	2.38	9.59	6.82

Table S4. Statistics of the simulated surface NO_2 against the surface measurements in March-May 2020. The unit for MB, MAB, and RMSE is ppbv. MB is based on the model values minus the observed ones.

	'England'	'Madrid'	'Po Valley'	'East Europe'	'Central'	'Northern Germany'	'Paris'	'Belgium/Holland'	'Rome/Milan'	'Barcelona'
Corr (prior)	0.26	0.65	0.38	0.31	0.18	0.42	0.75	0.56	0.32	0.85
Corr (post)	0.55	0.73	0.40	0.33	0.24	0.41	0.76	0.54	0.36	0.92
MB (prior)	-2.86	-2.45	-4.53	-4.33	-3.10	-3.78	-4.38	-0.82	-5.36	-3.40
MB (post)	-3.14	-1.72	-3.68	-3.72	-2.97	-4.65	-4.15	-1.14	-4.57	-2.61
MAB (prior)	2.95	2.45	4.53	4.39	3.43	4.14	4.38	1.50	5.36	3.40
MAB (post)	3.14	1.72	3.68	3.84	3.21	4.66	4.15	1.63	4.57	2.61
RMSE (prior)	3.38	2.66	4.83	4.95	4.31	4.55	4.65	1.96	5.75	3.65
RMSE (post)	3.48	1.99	4.07	4.44	3.99	5.16	4.43	2.12	4.98	2.78

References

Chan Miller, C., Jacob, D. J., Marais, E. A., Yu, K., Travis, K. R., Kim, P. S., Fisher, J. A., Zhu, L., Wolfe, G. M., Hanisco, T. F., Keutsch, F. N., Kaiser, J., Min, K.-E., Brown, S. S., Washenfelder, R. A., González Abad, G. and Chance, K.: Glyoxal yield from isoprene oxidation and relation to formaldehyde: chemical mechanism, constraints from SENEX aircraft observations, and interpretation of OMI satellite data, Atmospheric Chemistry and Physics, 17(14), 8725–8738, https://doi.org/10.5194/acp-17-8725-2017, 2017.

Copernicus Sentinel data processed by ESA, German Aerospace Center (DLR) (2019), Sentinel-5P TROPOMI Tropospheric Formaldehyde HCHO 1-Orbit L2 5.5km x 3.5km, Greenbelt, MD, USA, Goddard Earth Sciences Data and Information Services Center (GES DISC), Accessed: [Data Access Date], https://doi.org/10.5270/S5P-tjlxfd2

De Smedt, I., Theys, N., Yu, H., Danckaert, T., Lerot, C., Compernolle, S., Van Roozendael, M., Richter, A., Hilboll, A., Peters, E., Pedergnana, M., Loyola, D., Beirle, S., Wagner, T., Eskes, H., van Geffen, J., Boersma, K. F. and Veefkind, P.: Algorithm theoretical baseline for formaldehyde retrievals from S5P TROPOMI and from the QA4ECV project, Atmospheric Measurement Techniques, 11(4), 2395–2426, https://doi.org/10.5194/amt-11-2395-2018, 2018.

Karlsson, P. E., Ferm, M., Tømmervik, H., Hole, L. R., Pihl Karlsson, G., Ruoho-Airola, T., Aas, W., Hellsten, S., Akselsson, C., Mikkelsen, T. N. and Nihlgård, B.: Biomass burning in eastern Europe during spring 2006 caused high deposition of ammonium in northern Fennoscandia, Environmental Pollution, 176, 71–79, https://doi.org/10.1016/j.envpol.2012.12.006, 2013.

Lambert, J.-C., Compernole, S., Eichmann, K.-U., Graaf, M. de, Hubert, D., Keppens, A., Kleipool, Q., Langerock, B., Sha, M. K., Verhoelst, T., Wagner, T., Ahn, C., Argyrouli, A., Balis, D., Chan, K. L., Smedt, I. De, Eskes, H., Fjæraa, A. M., Garane, K., Gleason, J. F., Goutail, F., Granville, J., Hedelt, P., Heue, K.-P., Jaross, G., Koukouli, M., Landgraf, J., Lutz, R., Nanda, S., Niemejer, S., Pazmiño, A., Pinardi, G., Pommereau, J.-P., Richter, A., Rozemeijer, N., Sneep, M., Zweers, D. S., Theys, N., Tilstra, G., Torres, O., Valks, P., Vigouroux, C., Wang, P. and Weber, M.: Quarterly Validation Report of the Copernicus Sentinel-5 Precursor Operational Data Products #06: April 2018 - February 2020. http://www.tropomi.eu/sites/default/files/files/publicS5P-MPC-IASB-ROCVR-02.0.2-20190411_FINAL.pdf

Pusede, S. E., Steiner, A. L. and Cohen, R. C.: Temperature and Recent Trends in the Chemistry of Continental Surface Ozone, Chem. Rev., 115(10), 3898–3918, https://doi.org/10.1021/cr5006815, 2015.

Vigouroux, C., Langerock, B., Bauer Aquino, C. A., Blumenstock, T., Cheng, Z., De Mazière, M., De Smedt, I., Grutter, M., Hannigan, J. W., Jones, N., Kivi, R., Loyola, D., Lutsch, E., Mahieu, E., Makarova, M., Metzger, J.-M., Morino, I., Murata, I., Nagahama, T., Notholt, J., Ortega, I., Palm, M., Pinardi, G., Röhling, A., Smale, D., Stremme, W., Strong, K., Sussmann, R., Té, Y., van Roozendael, M., Wang, P. and Winkler, H.: TROPOMI–Sentinel-5 Precursor formaldehyde validation using an extensive network of ground-based Fourier-transform infrared stations, Atmospheric Measurement Techniques, 13(7), 3751–3767, https://doi.org/10.5194/amt-13-3751-2020, 2020.
